# The MdERF1B-miR482c-*MdCCR4-MdTLP1b* module regulates apple resistance to *Colletotrichum gloeosporioides*

**DOI:** 10.1093/hr/uhag199

**Published:** 2026-05-21

**Authors:** Baodong Zhang, Jialin Cong, Zongshan Zhou

**Affiliations:** Research Institute of Pomology, Chinese Academy of Agriculture Sciences, Xingcheng 125100, China; Research Institute of Pomology, Chinese Academy of Agriculture Sciences, Xingcheng 125100, China; Research Institute of Pomology, Chinese Academy of Agriculture Sciences, Xingcheng 125100, China

## Abstract

Glomerella leaf spot (GLS), incited by *Colletotrichum gloeosporioides*, poses a significant threat to apple (*Malus domestica*) production. microRNAs (miRNAs) are critical regulators of plant–pathogen crosstalk. However, the underlying mechanism through which miR482 regulates apple resistance to GLS remains unclear. Here, we show that overexpression of miR482c compromises resistance against *C. gloeosporioides*, while silencing miR482c confers resistance to *C. gloeosporioides*. 5′RACE assays in apple and co-expression assays in *Nicotiana benthamiana* confirmed that miR482c targets and cleaves the *MdCCR4* mRNA. Subsequent research revealed that overexpression of *MdCCR4* significantly enhances apple resistance to *C. gloeosporioides*. Notably, MdCCR4 physically interacts with the thaumatin-like protein MdTLP1b, which also positively regulates resistance to *C. gloeosporioides*. Additionally, MdERF1B was identified as an upstream regulator of miR482c through bioinformatics and Y1H screening, and was further confirmed to negatively regulate miR482c transcription by directly binding to its promoter. Meanwhile, *MdERF1B* overexpression enhances resistance to *C. gloeosporioides*, while silencing *MdERF1B* compromises resistance to *C. gloeosporioides*. Collectively, our findings indicate that the MdERF1B-miR482c-*MdCCR4-MdTLP1b* regulatory network is pivotal for apple defense against *C. gloeosporioides*, highlighting promising candidates for the genetic improvement of disease resistance.

## Introduction

Apple is a globally vital deciduous fruit crop, but its cultivation is frequently plagued by fungal diseases such as *Alternaria* leaf spot, Valsa canker, ring rot, and Glomerella leaf spot (GLS) [1–3].

GLS, incited by *Colletotrichum gloeosporioides*, stands out as a major threat, with outbreaks exacerbated by high temperature and humidity [4, 5]. Currently, control of GLS depends heavily on chemical treatments, raising concerns about environmental safety and public health. Thus, breeding resistant cultivars represents a more sustainable strategy. However, developing such cultivars requires a deep understanding of the molecular interplay between apple and *C. gloeosporioides*.

Noncoding RNAs (ncRNAs) serve as critical regulatory molecules that facilitate plant adaptation to fluctuating environmental stress [6]. In particular, microRNAs (miRNAs) fine-tune plant immunity by dampening the expression of defense-associated genes at the post-transcriptional level [7]. miR482, an miRNA closely associated with disease resistance, primarily participates in the interaction between plants and pathogens by negatively regulating NLR genes [8]. In potato, overexpression (OE) of miR482e triggers secondary tasiRNAs by targeting NBS-LRR, promotes sensitivity to *Verticillium dahliae* [9]. Similarly, in tomato, OE of miR482c suppresses NBS-LRR target genes and antioxidant enzyme activities, leading to reduced ROS scavenging capacity and exacerbated membrane lipid peroxidation, which ultimately enhances plant susceptibility to *Phytophthora infestans* [10]*.* Furthermore, multiregulatory analysis shows that poplar miR482a triggers phasiRNAs by targeting PHA7, thereby reducing ROS accumulation and enhancing susceptibility to the canker pathogen *Lonsdalea populi* [11]. Similarly, in apple, miR482b promotes susceptibility to *Alternaria alternata f. sp. mali* by targeting *MdTNL1* and initiating phasiRNA biogenesis to further repress immune responses [12]. Despite the well-documented negative regulatory role of the miR482 family across various species, the distinct contribution and underlying mechanism of miR482c in response to *C. gloeosporioides* represent a knowledge gap.

Plants orchestrate a robust immune response against pathogens by harnessing the induced upregulation of numerous defense-related genes [13]. Notably, pathogenesis-related (PR) genes constitute a major part of these genes, a group closely associated with systemic acquired resistance [14]. So far, researchers have identified 17 distinct PR gene families, such as thaumatin-like proteins [15]. Thaumatin-like proteins (TLPs), which constitute the PR5 family among the 17 identified PR proteins, combat fungal pathogens by suppressing both mycelial development and spore germination [16]. Heterologous expression of the rice *Ostlp* gene conferred resistance to cassava against infection by *C. gloeosporioides f. sp. manihotis* [17]. In wheat, by activating defense-associated enzymes and reactive oxygen species pathways, the OE of *TaTLP1* in wheat confers significant resistance against *Bipolaris sorokiniana* and *Puccinia triticina* [18]. In potato, OE of *CsTLP* significantly improved tolerance to *Macrophomina phaseolina* and *P. infestans* infection by activating intrinsic defense pathways involving the upregulation of *StPAL* and *StLOX* genes [19]. Notably, PR5 family proteins frequently serve as targets for pathogen-secreted effectors to modulate host immunity. Specifically, the interaction of PevD1 (a *V. dahliae* effector) with GhPR5 impairs antifungal activity in cotton [20]. Similarly, PR5 osmotin binds to BcIEB1 to modulate plant immunity against *Botrytis cinerea* [21]. In parallel with these findings, the effector BEC1054 from *Blumeria graminis* has also been reported to interact with PR5 proteins [22], further illustrating the prevalence of this pathogen strategy. Extensive studies have highlighted the significance of TLPs in fungal defense and their manipulation by effectors. However, the specific role of *TLP1b* in the resistance of apple (*Malus domestica*) against *C. gloeosporioides* remains largely unexplored.

Transcription factors (TFs) orchestrate complex biological networks by precisely modulating gene expression [23]. Among them, the ethylene response factor (ERF) family stands out as a critical class of transcription factors that exerts a pivotal function in regulating plant immunity and defense responses against pathogens [24]. Mechanistically, ERFs counteract pathogen infection through regulating the expression of PR genes as well as those involved in the biosynthesis of physical barriers and secondary metabolites [25]. For instance, OE of *MdERF11* boosts the transcription of salicylic acid-related genes (*EDS1*, *PAD4*) and PR genes (*PR1*, *PR5*), thereby enhancing resistance to apple ring rot [26]. Likewise, by regulating hormone signaling (salicylic acid and jasmonic acid), ectopic expression of *MdERF100* in Arabidopsis significantly improves defense against powdery mildew [27]. Furthermore, *MdERF114* strengthens resistance against *F. solani* by promoting physical barrier fortification via *PRX63*-mediated lignin deposition [28]. Thus, uncovering the precise mechanisms of specific ERF members during the apple–*C. gloeosporioides* interaction is a crucial research priority.

Our previous studies have demonstrated that miR482c is induced by *C. gloeosporioides*. In this study, we identified a novel regulatory module, MdERF1B-miR482c-*MdCCR4-MdTLP1b*, that orchestrates apple resistance to *C. gloeosporioides*. Our findings reveal that miR482c compromises resistance against GLS by post-transcriptionally silencing the protein kinase gene *MdCCR4*. Notably, MdCCR4 was found to physically interact with the thaumatin-like protein MdTLP1b, and both proteins positively regulate the immune to *C. gloeosporioides*. In addition, we show that the nucleus-localized transcription factor MdERF1B positively regulates defense against *C. gloeosporioides* and represses miR482c transcription by binding its promoter. Collectively, our findings elucidate the hierarchical regulation of the MdERF1B-miR482c-*MdCCR4*-*MdTLP1b* cascade, thus deepening our understanding of the apple–pathogen pathosystem and facilitating the genetic improvement of GLS resistance in breeding.

## Results

### miR482c directly cleaves protein kinase *MdCCR4* mRNA

As a deeply conserved lineage, miR482 is known to exist in 26 plant species, including tomato, grape, and apple (Fig. S1). miR482c was upregulated in response to *C. gloeosporioides* in the susceptible varieties ‘Royal Gala’ (‘GL-3’) through sRNA-seq in our previous study [29]. To understand the characteristics of the apple miR482 family, all mature sequences of apple miR482 from the miRBase database were downloaded and subjected to multiple sequence alignment, with the results shown in Fig. S2A. The alignment results showed significant sequence divergence among members of the apple miR482 family, while miR482b and miR482c exhibited high conservation. To investigate the genomic context of miR482c, we aligned its precursor sequence against the apple reference genome. Genomic mapping anchored the miR482c precursor to the 2 405 018–2 405 158 region of chromosome 13. Subsequent analysis of RNA secondary structure confirmed its ability to form a canonical stem-loop, exhibiting a minimal free energy of −52.9 kcal/mol (Fig. S2C). Following the retrieval of the miR482c promoter from the GDR database, putative cis-elements were predicted using the PlantCARE website. The presence of specific binding sites implies that miR482c expression is subject to regulation by phytohormones like abscisic acid, gibberellin, and salicylic acid, as well as general defense pathways (Fig. S2D).

We employed the psRNATarget online tool (https://www.zhaolab.org/psRNATarget/) applying standard settings to screen for candidate targets of miR482c. Serine/threonine protein kinase *MdCCR4* are predicted to be target genes of miR482c. To verify this prediction, 5′ rapid amplification of cDNA ends (RACE) assay was performed using total RNA extracted from apple leaves through HiScript-TS 5′/3′ RACE Kit. It was found that the 619–620 base site of *MdCCR4* can be cleaved by miR482c, suggesting that miR482c directly cleaves protein kinase *MdCCR4* mRNA. To further verify whether miR482c could interact with *MdCCR4*, *MdCCR4* was co-injected with 35S:miR482c into *N. benthamiana*. 35S:GUS was utilized as the positive control, while 35S:miR482c acted as the negative control (Fig. 1B). Histochemical GUS staining results exhibited no visible color in *N. benthamiana* expressing only 35S:miR482c. In contrast, leaves transformed with 35S:GUS or 35S:*MdCCR4*-GUS alone displayed intense blue staining. After co-injecting and expressing 35S:miR482c and 35S:*MdCCR4*-GUS in *N. benthamiana*, the leaves of tobacco plants co-expressing the genes showed significantly lighter pigmentation compared to those expressing only 35S:GUS or 35S:*MdCCR4*-GUS (Fig. 1C). Quantitative GUS fluorescence activity was detected in these plants, supporting the results of GUS histochemical staining (Fig. 1D). In conclusion, these results confirm that miR482c can target and cleave *MdCCR4* transcripts through complementary base pairing.

**Figure 1:**
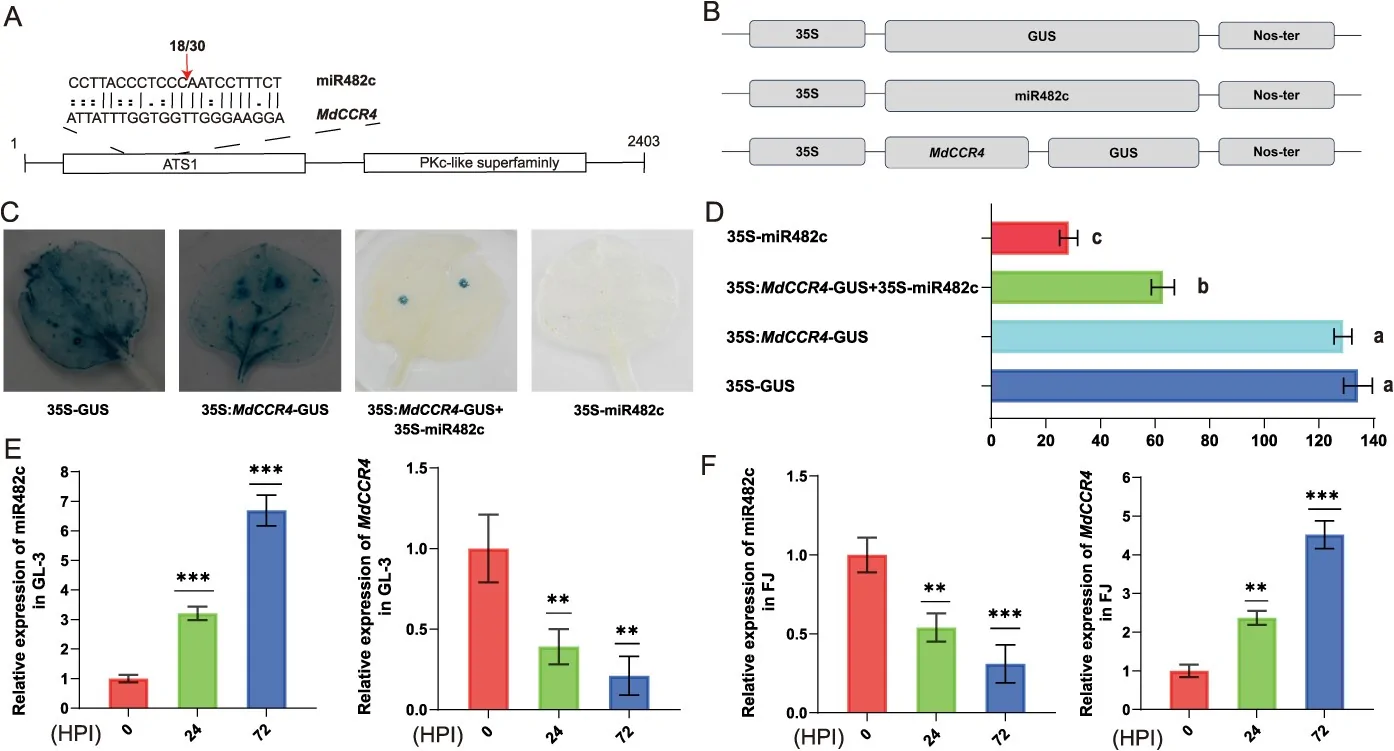
*MdCCR4* gene is targeted by miR482c. A. Identification of the cleavage sites of miR482c in apple using 5′ RACE technology. B. Schematic diagrams of constructs used in *N. benthamiana* co-transformation. C. Histochemical GUS assays were performed on *N. benthamiana* leaves 3 days after infiltration with the various constructs. The displayed images are representative of at least three independent experiments. D. Analysis of GUS activity in *N. benthamiana* leaves transiently expressing various constructs. E. The expression levels of miR482c and *MdCCR4* in the leaves of the susceptible variety ‘GL-3’ at different infection times were detected using RT-qPCR. F. Expression levels of miR482c and its target gene *MdCCR4* in the leaves of the disease-resistant variety ‘FJ’ at different infection times. Asterisks denote statistically significant differences (^*^P < 0.05, ^**^P < 0.01, ^***^P < 0.001). This was determined by one-way ANOVA followed by Tukey’s test.

To verify whether miR482c and *MdCCR4* respond to *C. gloeosporioides* infection, their expression profiles were monitored in ‘GL-3’ and ‘FJ’ leaves. RT-quantitative PCR (qPCR) assays revealed a marked induction of both pre-miR482c and mature miR482c at 24 and 72 HPI in the leaves of ‘GL-3’ (Figs 1E and S3A), while *MdCCR4* expression levels were markedly downregulated (Fig. 1E). In contrast, the ‘FJ’ cultivar exhibited a significant downregulation of pre-miR482c and miR482c (Figs 1F and S3B), which coincided with a robust upregulation of *MdCCR4* transcripts at 24 and 72 HPI (Fig. 1F).

### miR482c negatively regulates resistance to GLS in apples

Given that *C. gloeosporioides* infection triggers miR482c accumulation, we proposed that this miRNA is integral to the resistance response in apple. To verify this hypothesis, vectors for miR482c OE and silencing were constructed, as shown in Fig. S4. Transient transformation of apple leaves was performed with these vectors using *Agrobacterium*-mediated method, with OE-miR482c overexpressed in ‘FJ’ leaves and STTM-miR482c introduced into ‘GL-3’ leaves. At 4 days postinfiltration, the transcript abundance of miR482c was quantified via RT-qPCR. RT-qPCR analysis revealed a nearly 19-fold elevation of miR482c expression levels in OE-miR482c plants (Fig. 2A) and a 69% reduction in STTM-miR482c plants (Fig. 2D), indicating the successful generation of transient miR482c OE and silenced leaves.

**Figure 2:**
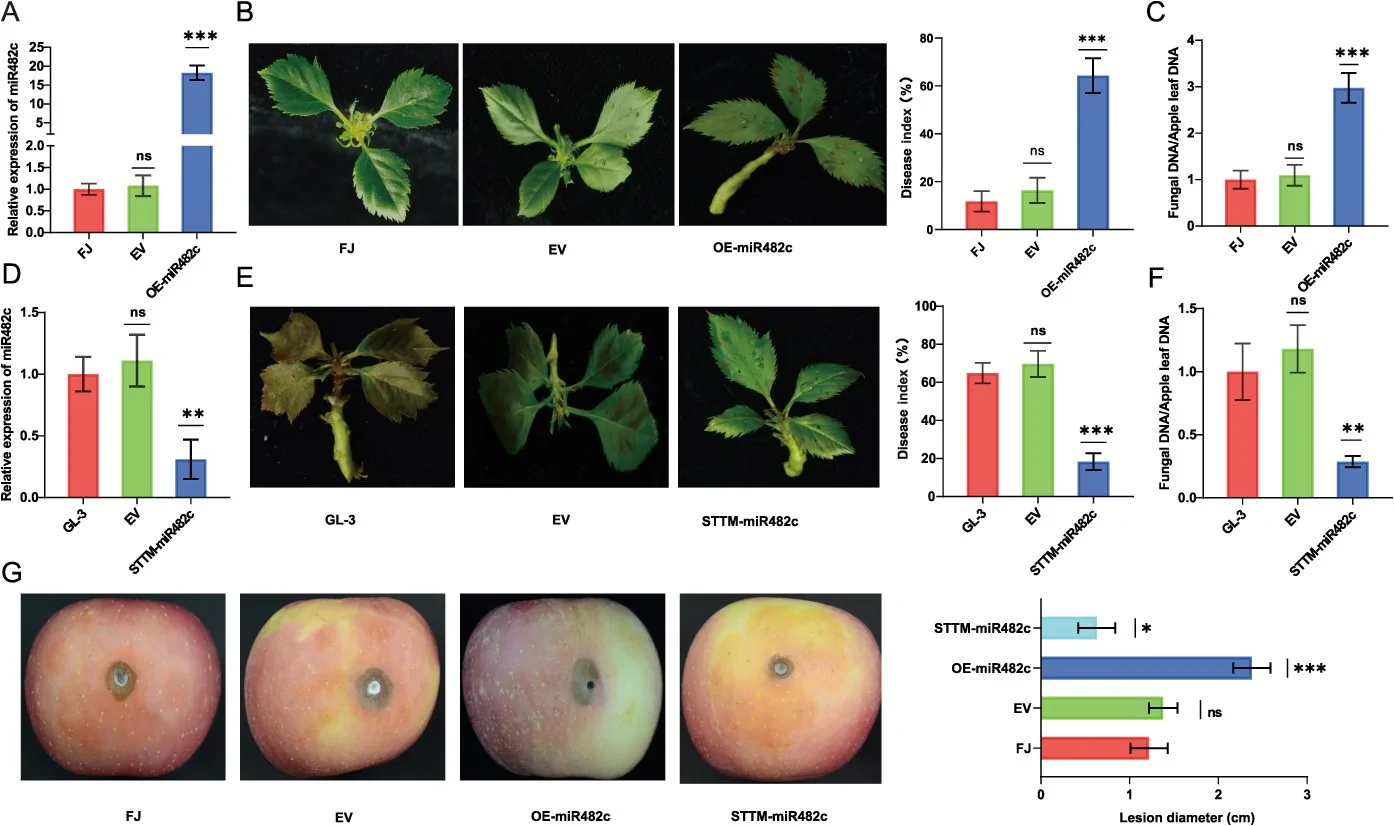
miR482c enhances susceptibility to GLS in apples. The expression level of miR482c in ‘FJ’, EV, and OE-miR482c plants was detected using RT-qPCR. B. Representative disease symptoms and statistical analysis of disease rates in ‘FJ’, EV, and OE-miR482c leaves inoculated with *C. gloeosporioides*. C. Relative biomass of *C. gloeosporioides* was examined by RT-qPCR in these leaves. D. The relative transcript abundance of miR482c in ‘GL-3’, EV, and STTM-miR482c plants was determined through RT-qPCR. Typical disease symptoms along with the statistical assessment of disease rates in ‘GL-3’, EV, and STTM-miR482c leaves challenged with *C. gloeosporioides* were shown in E. F. RT-qPCR analysis was performed to determine the relative biomass of *C. gloeosporioides* in the indicated plants. G. Representative images of disease symptoms and statistical analysis of disease rates in ‘FJ’, EV, OE-miR482c, and STTM-miR482c fruits inoculation with *C. gloeosporioides*. Data are presented as means ± SD from three independent biological replicates (*n* = 3). The asterisks indicate significant differences, determined by One-way ANOVA followed by Tukey’s test.

Subsequently, *C. gloeosporioides* (with a concentration of approximately 1 × 10^6^ conidia·ml^−1^) inoculation was carried out on ‘FJ’ leaves, as well as those expressing OE-miR482c or the empty vector (EV), to assess the function of miR482. The changes in lesions were observed 3 days after inoculation, the typical symptoms are shown in Fig. 2B. Exacerbated disease phenotypes were observed in miR482c-overexpressing plants, accompanied by a marked elevation in the disease index. Meanwhile, RT-qPCR detection revealed that the biomass of miR482c-overexpressing plants also significantly increased compared to EV plants (Fig. 2C). We continued to analyze the phenotypic changes in STTM-miR482c plants after inoculation with *C. gloeosporioides*. Compared to EV and ‘GL-3’ plants, STTM-miR482c plants exhibited milder disease symptoms and a smaller disease index at 3 d after infection by *C. gloeosporioides* (Fig. 2E). A significant decrease in biomass was detected in STTM-miR482c leaves compared to EV leaves using RT-qPCR (Fig. 2F). In addition, because the ‘GL-3’ is exclusively maintained as tissue-cultured plantlets in our facility, we further injected the OE-miR482c and STTM-miR482c vectors into ‘FJ’ fruits and inoculated them with *C. gloeosporioides*. The size of fruit necrotic lesions was observed 3 days after inoculation. As shown in Fig. 2G, fruits overexpressing miR482c exhibited longer lesions, whereas fruits with silenced miR482c showed smaller lesions. Collectively, these findings suggest that miR482c attenuates defense against *C. gloeosporioides*.

According to previous research reports [8], miR482 typically targets and cleaves NLR genes in response to pathogen infection. In this study, we investigated the transcript abundance of PR genes, given that NLR receptors facilitate their regulation by activating transcription factors like WRKY [30, 31]. Particularly, the transcript abundance of *MdPR1, MdPR5, and MdPR10* was quantified in OE-miR482c and STTM-miR482c plants via RT-qPCR. As Fig. S5 shown, expression levels of *MdPR1*, *MdPR5*, and *MdPR10* in OE-miR482c plants were apparently reduced, while they exhibited an opposite expression trend in STTM-miR482c plants. Taken together, our findings establish that the OE of miR482c downregulates *MdPR1, MdPR5, and MdPR10*, consequently facilitating *C. gloeosporioides* infection.

### *MdCCR4* positively regulates tolerance of apple to *C. gloeosporioides*

To further understand how *MdCCR4* contributes to apple defense against *C. gloeosporioides*, we cloned the complete coding sequence (CDS) of *MdCCR4* into the pRI101-GFP plasmid (Fig. S6A). Subsequently, the plasmid transiently expressed in ‘GL-3’ through *Agrobacterium*-mediated infiltration, with WT and EV plants serving as controls. RT-qPCR analysis verified robust *MdCCR4* expression 4 days after transformation (Fig. 3A). Furthermore, the successful translation and accumulation of the MdCCR4 protein in the transiently transformed leaves were confirmed by western blot analysis using an anti-GFP antibody (Fig. S7). Following inoculation with *C. gloeosporioides*, the OE-*MdCCR4* plants displayed enhanced resistance, characterized by smaller lesion areas and a reduced disease index compared to the severe browning observed in the control plants (‘GL-3’ and EV) (Fig. 3B). Furthermore, analysis of fungal growth revealed a marked reduction in the biomass of *C. gloeosporioides* in the OE-*MdCCR4* plants compared to controls (Fig. 3C).

**Figure 3:**
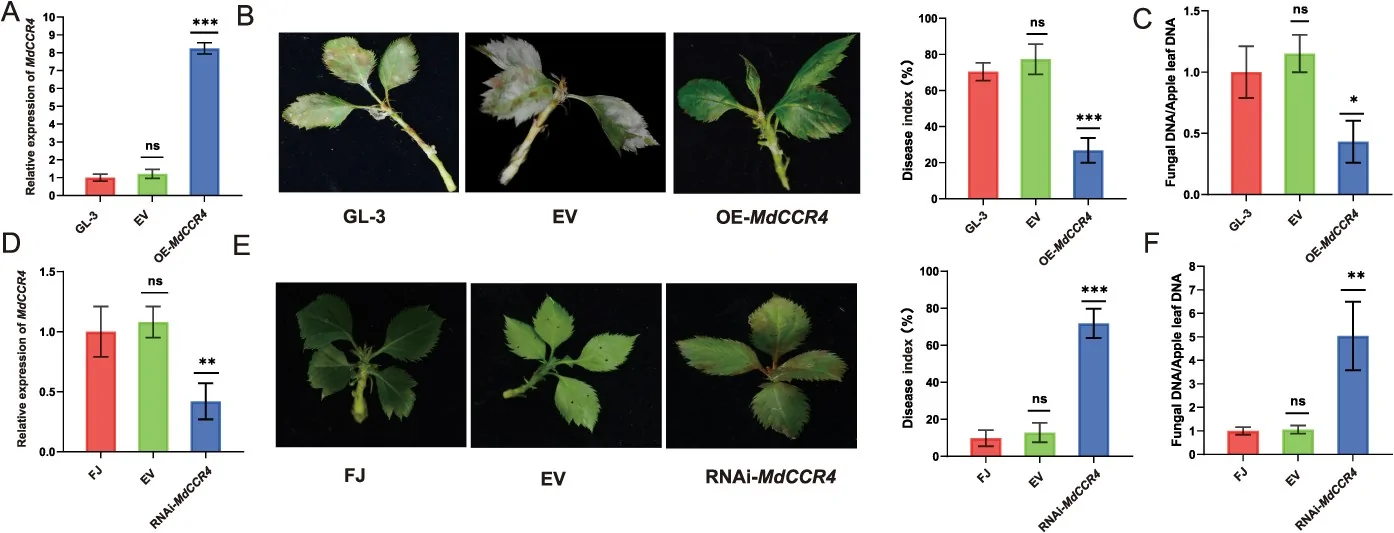
*MdCCR4* enhances resistance to GLS in apples. A. Quantitative real-time PCR was utilized to determine the expression level of *MdCCR4* across ‘GL-3’, EV, and OE-*MdCCR4* leaves. B. Phenotype and disease index of *MdCCR4*-overexpressing leaves three days after inoculation with *C. gloeosporioides.* C. Quantification of *C. gloeosporioides* growth on infected apple leaves non-infiltrated or infiltrated with different constructs. D. The amounts of *MdCCR4* were measured in ‘FJ’, EV, and RNAi-*MdCCR4* leaves through RT-qPCR. E. Disease symptoms and disease index on representative leaves of ‘FJ’, EV, and RNAi-*MdCCR4* following inoculation with *C. gloeosporioides*. F. Relative fungal biomass of *C. gloeosporioides* on ‘FJ’, EV, and RNAi-*MdCCR4*. The asterisks indicate significant differences, determined by one-way ANOVA followed by Tukey’s test.

To investigate the loss-of-function phenotype, we silenced endogenous *MdCCR4* using an RNA interference (RNAi) strategy (Fig. S6B). We performed *Agrobacterium*-mediated infiltration to deliver the RNAi-*MdCCR4* construct into ‘FJ’ leaves. Silencing efficiency of *MdCCR4* in the transiently transformed leaves was confirmed by RT-qPCR (Fig. 3D). To determine how the downregulation of *MdCCR4* influences disease resistance, we inoculated these leaves with *C. gloeosporioides*. In sharp contrast to the OE-*MdCCR4* plants, RNAi-*MdCCR4* leaves displayed rapidly expanding brown spots and a significantly higher disease index compared to ‘FJ’ and EV leaves (Fig. 3E). Consistent with these severe symptoms, fungal biomass increased meaningfully in RNAi-*MdCCR4* leaves (Fig. 3F). Taken together, these data indicate that *MdCCR4* positively regulates apple resistance to *C. gloeosporioides*.

### MdCCR4 physically interacts with MdTLP1b

Having established that *MdCCR4* promotes apple defense, we next investigated its downstream mechanisms. Sequence annotation identified *MdCCR4* as a putative serine/threonine protein kinase (Fig. 1A). Subcellular localization assays using *MdCCR4*-GFP in *N. benthamiana* revealed specific signal accumulation at both the plasma membrane and the nucleus (Fig. S8). To identify interacting partners within these compartments, we utilized an AI-driven virtual screening approach as a targeted alternative to conventional library screening. Through Hdock screening and further AlphaFold3 prediction screening, we selected the MD08G1011900 protein of interest from the top 10 scoring proteins as our research subject (Table S1). To determine the gene sequence, PCR amplification was performed using apple cDNA as the template to obtain the *TLP1b* (LOC103410424) gene. Sequence analysis revealed that *MdTLP1b* possesses a coding region of 966 bp, corresponding to 321 amino acid residues.

To verify interplay between MdCCR4 and MdTLP1b, A Y2H assay was implemented. *MdCCR4* was inserted into pGBKT7 (pGBKT7-*MdCCR4*) and *MdTLP1b* was inserted into pGADT7 vector (pGADT7-*MdTLP1b*), respectively. Next, plasmids were simultaneously introduced into Y2HGold yeast cells. Protein–protein interplay in Y2H cell was assessed by monitoring cell viability and chromogenic reactions. Yeast strains co-expressing pGBKT7-*MdCCR4* and pGADT7-*MdTLP1b* exhibited normal growth on the selective medium (SD/-Trp/-Leu/-His/-Ade). Furthermore, these colonies developed a blue color in the presence of X-α-Gal (Fig. 4B). These data provide evidence for the physical binding of MdCCR4 to MdTLP1b.

**Figure 4:**
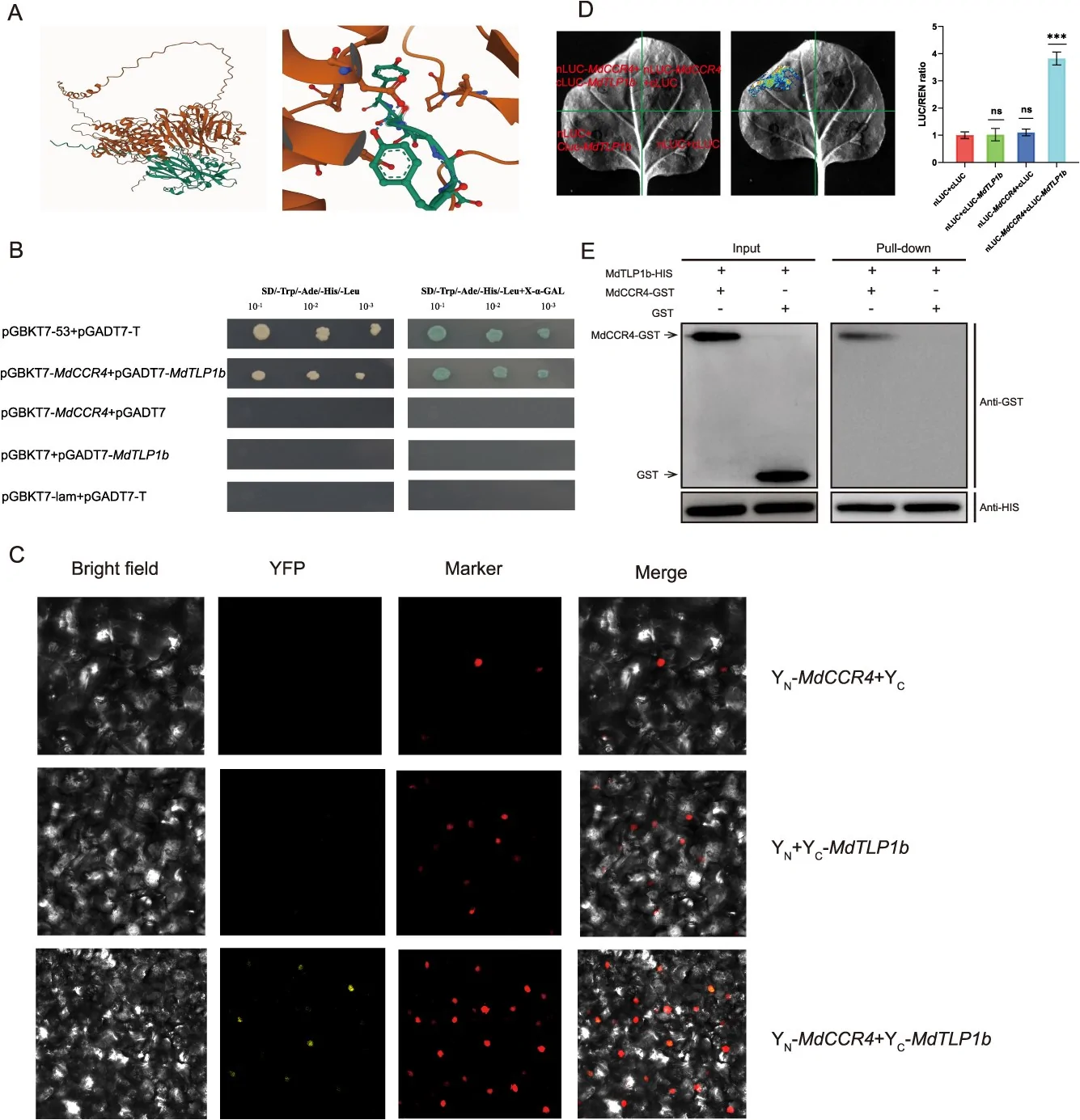
MdCCR4 physically interacts with MdTLP1b *in vivo* and *in vitro*. The molecular docking works were performed by AlphaFold Server (https://alphafoldserver.com/) to verify the potential interaction between MdCCR4 and MdTLP1b. B. Yeast two-hybrid (Y2H) assay showing the interaction between MdCCR4 and MdTLP1b. pGBKT7–53 + pGADT7-T served as positive control, while pGBKT7-lam + pGADT7-T act as negative control. C. Assay of bimolecular fluorescence complementation (BiFC) confirmed MdCCR4 interacts with MdTLP1b *in vivo*. D. Split-luciferase complementation assay showing the interaction MdCCR4 and MdTLP1b. E. Pull-down assay confirmed MdCCR4 interacts with MdTLP1b *in vitro*.

To substantiate the physical association between MdCCR4 and MdTLP1b *in vivo*, we utilized both bimolecular fluorescence complementation (BiFC) and luciferase complementation imaging (LCI) assay. For BiFC, *Nicotiana benthamiana* leaves co-expressing *MdCCR4* and *MdTLP1b* displayed strong nuclear YFP fluorescence. In contrast, the negative control groups failed to exhibit any fluorescent signal (Fig. 4C). This interaction was further corroborated by LCI assays. Strong luminescence was exclusively detected in leaf regions co-expressing nLUC-*MdCCR4* and cLUC-*MdTLP1b*, whereas control areas remained nonluminescent. Subsequent quantification revealed markedly higher luciferase activity in samples co-expressing nLUC-*MdCCR4* and cLUC-*MdTLP1b* compared to controls. These data collectively confirm that MdCCR4 physically interacts with MdTLP1b in plant cells.

We further substantiated this interaction in a pull-down approach. Recombinant *MdTLP1b*-HIS and *MdCCR4*-GST plasmids were expressed in *Escherichia coli* BL21 (DE3) following IPTG induction. Subsequently, *MdTLP1b*-HIS was mixed with *MdCCR4*-GST or a GST negative control, followed by capture on Ni-NTA beads. The assay revealed that *MdTLP1b*-HIS was specifically pulled down by *MdCCR4*-GST, but not by the GST control. In summary, our data from both cellular and cell-free systems confirm the direct interaction between MdCCR4 and MdTLP1b.

### The overexpression of *MdTLP1b* enhances apple resistance to *C. gloeosporioides*

Considering the established physical interplay between MdCCR4 and MdTLP1b, we investigated whether *MdTLP1b* also contributes to apple immunity against *C. gloeosporioides*. We first monitored *MdTLP1b* transcript levels in resistant and susceptible varieties using RT-qPCR. Notably, *C. gloeosporioides* infection triggers a rapid accumulation of *MdTLP1b* mRNA specifically in the resistant ‘FJ’ cultivar (Fig. S9), implying *MdTLP1b* is crucial for disease resistance. To assess the validity of this prediction, we generated OE and RNAi silencing constructs targeting *MdTLP1b*. These vectors were transiently delivered into ‘GL-3’ and ‘FJ’ leaves, respectively, via *Agrobacterium*-mediated method. Subsequent RT-qPCR validation 4 days postinfiltration confirmed effective manipulation of gene expression: OE-*MdTLP1b* leaves displayed markedly elevated transcript levels relative to EV controls (Fig. 5A), whereas the RNAi-*MdTLP1b* samples showed substantially suppressed *MdTLP1b* transcript abundance (Fig. 5E). Consistently, western blot analysis further verified that the MdTLP1b fusion protein was successfully expressed in these OE-MdTLP1b leaves at the translational level (Fig. S10).

**Figure 5:**
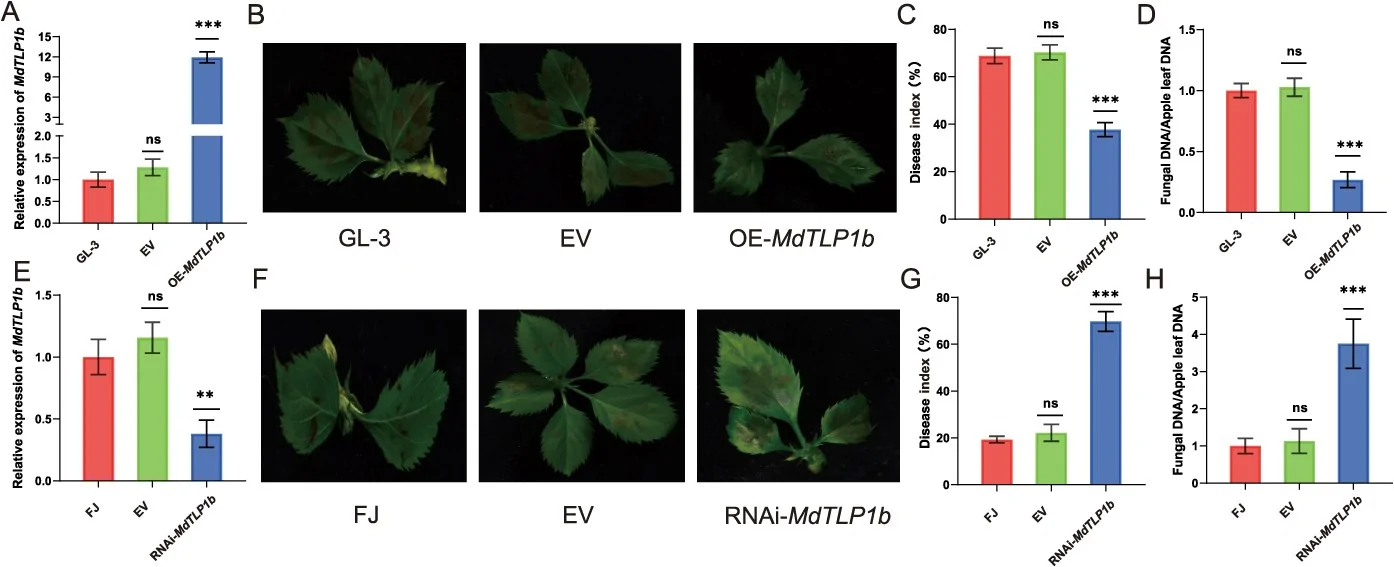
*MdTLP1b* confers resistance of apple to *C. gloeosporioides*. A. mRNA amounts of *MdTLP1b* in ‘GL-3’, EV, and OE-*MdTLP1b* leaves were examined using RT-qPCR. B. Disease symptoms on ‘GL-3’, EV, and OE-*MdTLP1b* leaves 3 days postinoculation (DPI) with *C. gloeosporioides*. And disease index (C) analysis of these leaves was conducted. D. Relative fungal biomass of *C. gloeosporioides* on the inoculated leaves of these plants. The fungal biomass was shown as the ratio of DNA levels of *C. gloeosporioides Tubulin* against DNA levels of apple *MdActin*. E. Transcript levels of *MdTLP1b* in ‘FJ’, EV, and RNAi-*MdTLP1b* plants. Silencing of *MdTLP1b* in apple leaves leads to increased susceptibility against *C. gloeosporioides* in ‘FJ’, EV, and RNAi-*MdTLP1b* plants, and typical disease symptoms were shown in (F). The disease index (G) was measured at 72 HPI. H. Comparative analysis of fungal biomass in ‘FJ’, EV, and RNAi-*MdTLP1b* following *C. gloeosporioides* inoculation. The asterisks indicate significant differences, determined by one-way ANOVA followed by Tukey’s test.

Next, inoculation with *C. gloeosporioides* was performed on the transiently transformed leaves to assess disease resistance. Overexpression of *MdTLP1b* significantly attenuated disease symptoms and decreased the disease index relative to controls (Fig. 5B and C). Conversely, silencing *MdTLP1b* in the resistant ‘FJ’ cultivar compromised its resistance, leading to more severe lesions and a higher disease index than in the ‘FJ’ and EV controls (Fig. 5F and G). Consistent with the phenotypic observations, the OE-*MdTLP1b* leaves exhibited significantly inhibited *C. gloeosporioides* growth, confirmed by a reduction in fungal biomass quantified via RT-qPCR (Fig. 5D) but promoted *C. gloeosporioides* growth in the RNAi-*MdTLP1b* leaves (Fig. 5H). Collectively, our data demonstrate that *MdTLP1b* positively regulates apple immunity against *C. gloeosporioides*.

Given that MdTLP1b physically associates with MdCCR4 and positively regulates apple immunity against *C. gloeosporioides*, we explored whether *MdCCR4* influences *MdTLP1b* transcription. In apple leaves overexpressing *MdCCR4*, we observed a marked elevation in *MdTLP1b* transcript abundance compared to vector controls. Conversely, silencing *MdCCR4* in the resistant cultivar ‘FJ’ led to a significant downregulation of *MdTLP1b* expression relative to the controls (Fig. S19E). Collectively, these findings provide evidence that *MdCCR4* promotes *MdTLP1b* expression, implying that *MdTLP1b* functions downstream of *MdCCR4* to confer resistance against *C. gloeosporioides*.

### MdERF1B represses miR482c promoter activity

Similar to protein-coding genes, MIR gene transcription is controlled by transcription factors and specific DNA motifs within their promoter regions [32]. To investigate the transcriptional regulation of miR482c, we cloned a 2.0-kb promoter region upstream of its precursor sequence (Fig. S11). Using a GUS reporter assay in ‘GL-3’ leaves, we evaluated promoter activity in response to *C. gloeosporioides* infection. Histochemical staining revealed intense blue signals in infected leaves compared to uninoculated controls (Fig. S12A). Quantitative assays corroborated these findings, showing significantly elevated GUS activity upon infection (Fig. S12B), indicating that the miR482c promoter is induced upon *C. gloeosporioides* infection. Furthermore, to identify potential transcription factors (TFs) that regulate miR482c expression during *C. gloeosporioides* infection, we predicted TF binding sites in the miR482c promoter using PlantTFDB (https://planttfdb.gao-lab.org/prediction.php). The results showed that members of 25 TF families potentially bind to this region and participate in the regulation of miR482c (Fig. S13).

Considering the established contribution of ERFs to apple immunity against *C. gloeosporioides*, relevant ERF transcription factors were selected as the research subjects. Subsequently, to identify specific ERF transcription factors associate with the miR482c promoter, we employed a yeast one-hybrid (Y1H) screening system. Y1HGold strain was simultaneously transformed with the bait plasmid (Pro_miR482c_-pAbAi) and various ERF prey constructs (Fig.S14). For quality control, the interaction between P53-pAbAi and Rec53-pGADT7 was used as a positive standard, while the empty pGADT7 vector paired with the bait served as a negative check. Screening results revealed that yeast cells carrying *MdERF1B*-pGADT7 exclusively survived on the selective medium (SD/-Leu supplemented with 500 ng/ml AbA), whereas cells expressing other ERF candidates failed to proliferate (Fig. 6A). This result indicated that MdERF1B bound the miR482c promoter in yeast.

**Figure 6:**
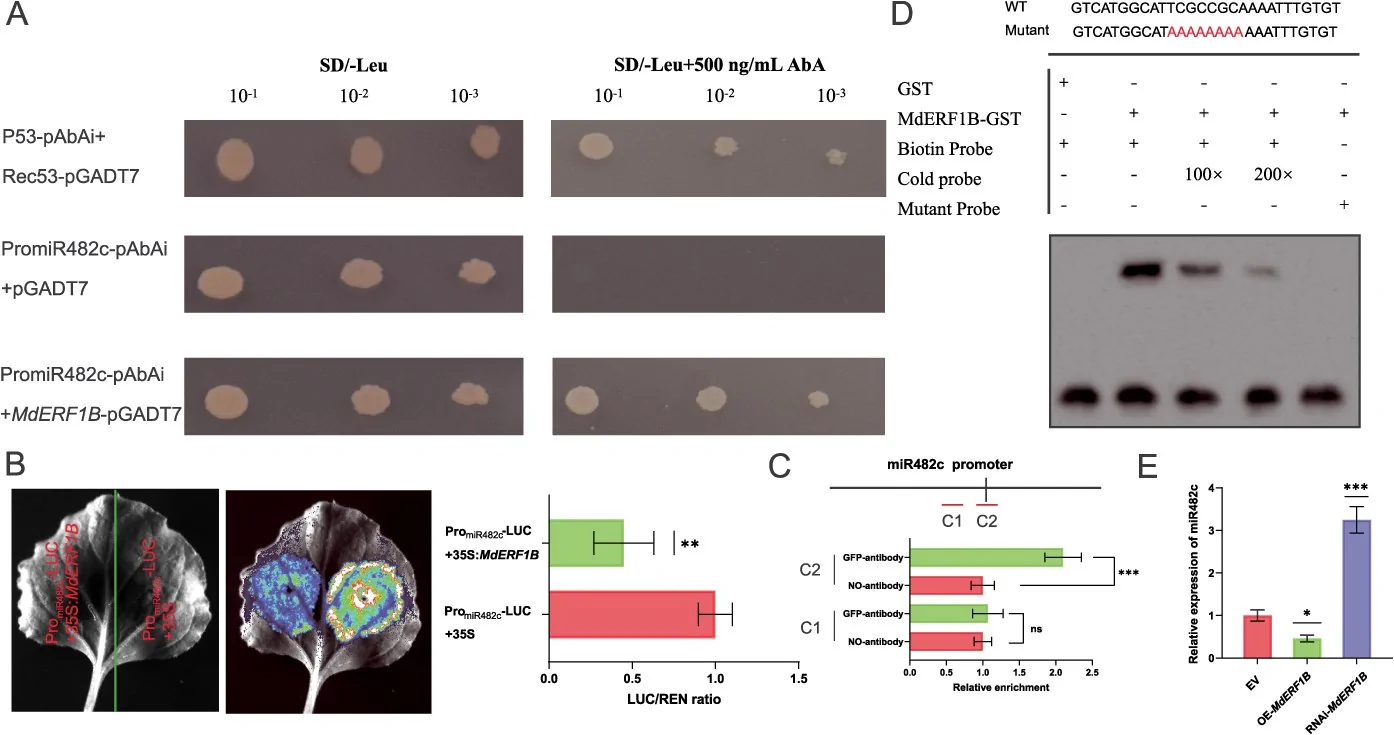
MdERF1B directly binds to the miR482c promoter and represses its transcription. Y1H assay of *MdERF1B* binding to the miR482c promoter fragments. Positive control: P53-pAbAi+Rec53-pGADT7; negative control: Pro_miR482c_-pAbAi+pGADT7. B. Dual-luciferase reporter assay in *N. benthamiana* leaves. The representative images and relative LUC/REN ratio indicate that *MdERF1B* represses the promoter activity of miR482c. C. ChIP-qPCR analysis confirmed MdERF1B bind to the miR482c promoter *in vivo*. The GCC-box binding site is present in C2 but absent in C1. D. Electrophoretic mobility shift assay (EMSA) showed the binding of MdERF1B to the miR482c promoter *in vitro*. E. The expression levels of miR482c were determined in OE-*MdERF1B* and RNAi-*MdERF1B* plants. The asterisks indicate significant differences, determined by One-way ANOVA followed by Tukey’s test.

To further validate the binding of MdERF1B to the miR482c promoter *in vivo*, we performed a ChIP-qPCR assay. As shown in Fig. 6C, in the negative control region (C1), no significant difference in DNA enrichment was observed between the GFP-antibody and the No-antibody control. However, in the specific promoter fragment (C2) harboring the binding motif, the enrichment level using the GFP-antibody was significantly higher than that of the No-antibody control. It became apparent that MdERF1B associated with miR482c promoter in apple calli. Dual-luciferase reporter analysis was carried out in *N. benthamiana* to verify the regulation of miR482c by MdERF1B. Specifically, we co-infiltrated the promiR482c:LUC reporter alongside the 35S:*MdERF1B* effector or an EV control. We noticed a substantial attenuation of luminescence intensity in leaves harboring *MdERF1B* compared to the control. Consistent with this observation, quantitative assays demonstrated a significantly lower LUC/REN ratio in leaves co-expressing *MdERF1B* compared to the control (Fig. 6B), signifying the transcription of miR482c is inhibited by MdERF1B in *N. benthamiana*.

To further verify MdERF1B directly binding to miR482c promoter, we employed an electrophoretic mobility shift (EMSA) to verify this interaction. EMSA results presented in Fig. 6D revealed that the MdERF1B-GST fusion protein formed a stable DNA-protein complex with the biotinylated wild-type probe, resulting in a retarded band, whereas the GST control did not. Crucially, this interaction proved to be specific: the shifted signal was gradually diminished by adding an excess of unlabeled cold probes but was undetectable when using a mutant probe lacking the core GC-rich motif. Our analysis revealed that MdERF1B is associated with the miR482c promoter. In addition, we verified through molecular docking that MdERF1B associated with miR482c promoter (Fig. S15).

Finally, we quantified the transcript abundance of miR482c in the OE-*MdERF1B* and RNAi-*MdERF1B* plants via RT-qPCR. The results revealed a distinct inverse correlation: miR482c expression was significantly downregulated in the OE-*MdERF1B* leaves, whereas it was markedly elevated in the RNAi-*MdERF1B* leaves (Fig. 6E). Combined with the results above, we conclude that MdERF1B directly targets the miR482c promoter to negatively regulate its transcription.

### The transcription factor MdERF1B enhances resistance to *C. gloeosporioides* infection

To examine the intracellular distribution of *MdERF1B*, we cloned its complete open reading frame (lacking the stop codon) into the PRI101-GFP plasmid. Following transient expression in *N. benthamiana* leaves, confocal imaging showed that the free GFP control displayed a diffuse signal throughout the cell. In contrast, the MdERF1B-GFP fusion protein accumulated specifically within the nucleus, overlapping perfectly with the nuclear marker (Fig. 7A). These observations establish that MdERF1B resides in the nucleus.

**Figure 7:**
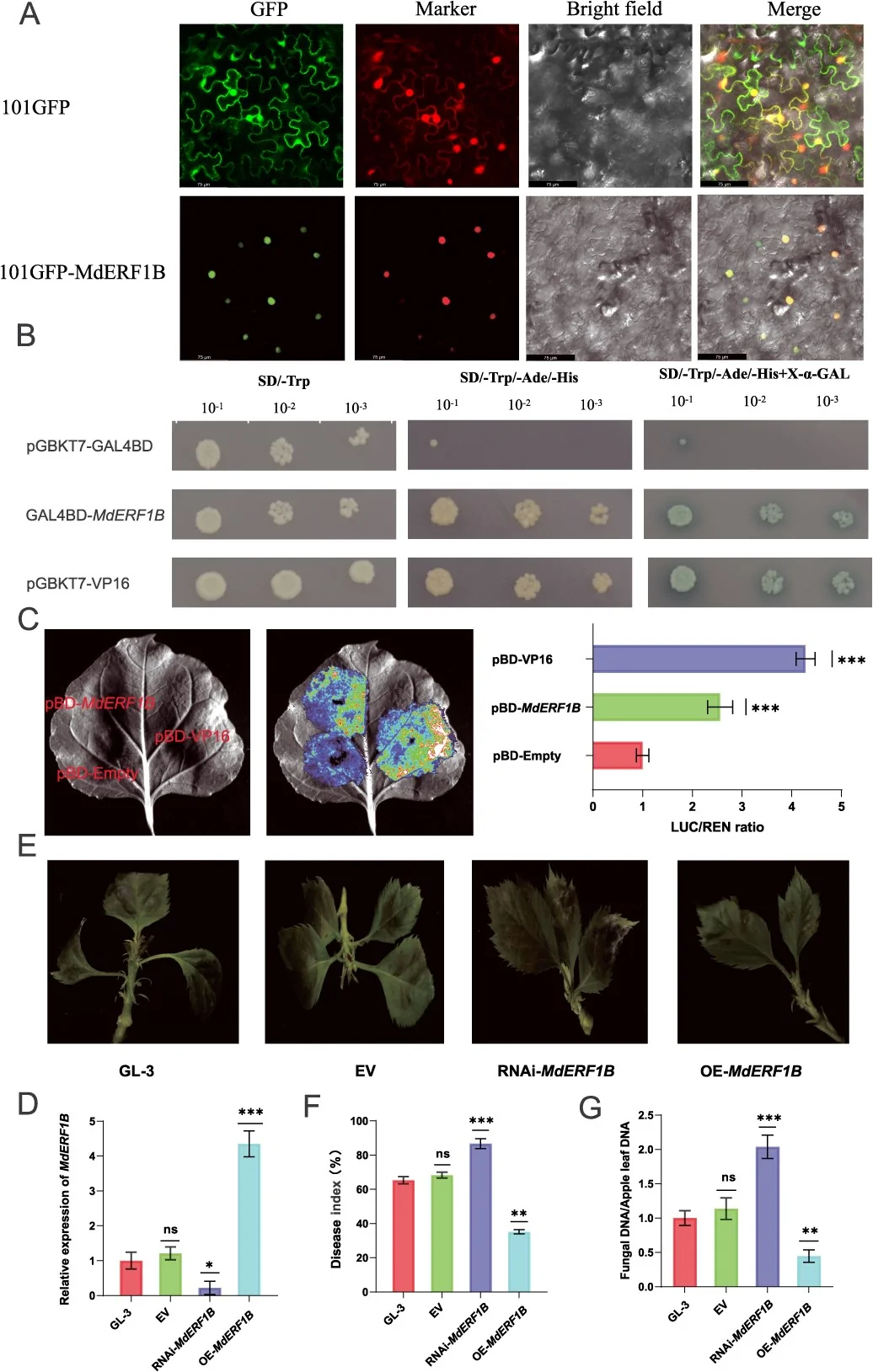
Subcellular localization, transcriptional activation activity of MdERF1B and its impact on resistance to *C. gloeosporioides*. Subcellular localization of *MdERF1B* in *N. benthamiana* leaves. The GFP signal (green) colocalizes with the nuclear marker (red). B. Transcriptional activation analysis of *MdERF1B* in yeast cells. C. Transcriptional activation activity of *MdERF1B* assessed by a dual-luciferase reporter system in *N. benthamiana* leaves. D. Quantitative real-time PCR was utilized to determine the expression level of *MdERF1B* across ‘GL-3’, EV, OE-*MdERF1B*, and RNAi-*MdERF1B* leaves. Phenotypes (E) analysis of plants overexpressing *MdERF1B* (OE-*MdERF1B*), silencing *MdERF1B* (RNAi-*MdERF1B*), and control plants (‘GL-3’ and EV) was observed, and the disease index (F) of these plants were measured at 72 HPI. G. Quantification of *C. gloeosporioides* relative biomass in these plants was monitored through RT-qPCR analysis.

To assess the transactivation potential of MdERF1B, we employed a yeast-based system. The CDS of *MdERF1B* was cloned into pGBKT7 to generate the GAL4BD-*MdERF1B* fusion construct, with empty pGBKT7 and VP16 serving as negative and positive controls, respectively. We observed that yeast cells harboring GAL4BD-*MdERF1B* were able to proliferate on triple-dropout medium (SD/-Trp/-Ade/-His) and developed a blue color upon X-α-Gal treatment (Fig. 7B). These phenotypes confirm that MdERF1B possesses intrinsic transcriptional activation ability. Dual-luciferase reporter analysis was employed in *N. benthamiana* leaves to confirm the transcriptional activity in plant cells. The schematic diagrams of the constructed vectors are presented in Fig. S16. Compared to the negative control, leaves expressing pBD-*MdERF1B* exhibited significantly stronger LUC intensity. This was corroborated by quantitative analysis, which revealed a significantly elevated LUC/REN ratio in the pBD-*MdERF1B* expressing tissues (Fig. 7C), confirming that MdERF1B possesses transcriptional activation activity *in vivo*.

To explore potential function of *MdERF1B* in GLS resistance, we first sought to understand the contribution of *MdERF1B* to GLS immunity by comparing its mRNA levels between resistant and susceptible apple varieties via RT-qPCR. Notably, infection with *C. gloeosporioides* triggered a distinct accumulation of *MdERF1B* transcripts in the resistant variety ‘FJ’ (Fig. S17). We next constructed *MdERF1B* OE and RNAi vectors and transiently delivered into ‘GL-3’ leaves mediated by *Agrobacterium*. In contrast to ‘GL-3’ and EV controls, transcript levels of *MdERF1B* were markedly elevated in the OE-*MdERF1B* leaves but substantially attenuated in the RNAi-*MdERF1B* leaves, as verified by RT-qPCR (Fig. 7D). Moreover, immunoblotting results confirmed the specific accumulation of the MdERF1B protein in the OE-MdERF1B apple leaves (Fig. S18). Subsequently, *C. gloeosporioides* inoculation was carried out on these treated leaves. The RNAi-*MdERF1B* leaves phenotypically developed aggravated disease symptoms characterized by expansive lesion areas, whereas the OE-*MdERF1B* leaves displayed significantly alleviated symptoms relative to the controls (Fig. 7E). These visual observations were substantiated by the disease index, which was notably elevated in the RNAi-*MdERF1B* leaves but suppressed in the OE-*MdERF1B* leaves (Fig. 7F). A similar pattern was observed regarding fungal growth: OE-*MdERF1B* plants harbored significantly less pathogen DNA, while RNAi-*MdERF1B* plants supported greater fungal colonization (Fig. 7G). Current findings indicate that *MdERF1B* contributes significantly to the defense of apple against *C. gloeosporioides.*

To validate the sequential regulatory cascade of the MdERF1B-miR482c-*MdCCR4*-*MdTLP1b* module, we assessed the transcript abundance of these downstream targets in transiently transformed apple leaves. RT-qPCR results showed that the transient OE of *MdERF1B* significantly upregulated the expression of *MdCCR4* and *MdTLP1b*, whereas their expression was markedly reduced in RNAi-*MdERF1B* leaves (Fig. S19A and C). Consistent with the negative regulation of miR482c by *MdERF1B*, the transient OE of miR482c significantly repressed the transcription of *MdCCR4* and *MdTLP1b*. In contrast, silencing miR482c (STTM-miR482c) resulted in the accumulation of both *MdCCR4* and *MdTLP1b* transcripts (Fig. S19B and D). Coupled with our earlier observation that *MdCCR4* positively dictates *MdTLP1b* expression (Fig. S19E), these findings suggested that upon *C. gloeosporioides* infection, *MdERF1B* promoted the expression of *MdCCR4* by repressing the transcription of miR482c and ultimately led to the accumulation of *MdTLP1b* in apple, thereby promoting a robust defense response against the fungal pathogen.

## Discussion

Glomerella leaf spot (GLS), primarily incited by *Colletotrichum* species such as *C. gloeosporioides* and *C. fructicola*, is a highly destructive fungal diseases that severely impacts apple (*M. domestica*) cultivation [33, 34]. The disease leads to severe defoliation and fruit necrosis, resulting in substantial economic losses [35]. Recent studies have demonstrated that the resistance to *C. gloeosporioides* can be regulated by multiple immune pathways. In the realm of transcriptional regulation, emerging studies have highlighted the contrasting functions of WRKY transcription factors in modulating defense against *C. gloeosporioides*. For example, *MdWRKY100* confers tolerance to *C. gloeosporioides* via the activation of SA-related genes *MdPAL1* and resistance gene *MdRPM1*, while VQ protein MdVQ37 physically interacts with MdWRKY100 to repress its transcriptional activity [36, 37]. Conversely, *MdWRKY17* attenuates resistance to *C. gloeosporioides* by activating the salicylic acid degradation gene *MdDMR6*, leading to a reduction in SA levels and promoting *C. gloeosporioides* infection. Similarly, *MdWRKY71* upregulates the SA degradation genes *MdDLO1,* thereby enhancing susceptibility to *C. gloeosporioides* [38, 39]. In addition, OE of *MdIPT8* confer resistance by elevating endogenous cytokinin levels [40], while miR7125-*MdARF1* module improves apple defense against *C. gloeosporioides* through promoting MdMYB46-mediated lignin synthesis [41]. The m^6^A reader protein MhYTP2 negatively modulates tolerance to *C. gloeosporioides* via physical association with the *MdRGA2L* transcript and the acceleration of its decay [42]. Exogenous chitosan enhances apple resistance to *C. gloeosporioides* by activating antioxidant defenses and salicylic acid-mediated immunity [43]. However, the molecular mechanisms by which ethylene response factors (ERFs) coordinate with miRNAs to regulate defense responses against *C. gloeosporioides* have not been fully characterized. In this study, we identified the MdERF1B-miR482c-*MdCCR4*-*MdTLP1b* module as a key regulator of apple resistance to *C. gloeosporioides* (Fig. 8). We demonstrate that MdERF1B inhibits the expression of miR482c, thereby relieving the repression of *MdCCR4*. Subsequently, accumulated MdCCR4 interacts with MdTLP1b to positively regulate the immune response (Fig. 4). Collectively, this multilevel cascade offers new insights into apple–pathogen interactions and valuable targets for resistance breeding.

**Figure 8:**
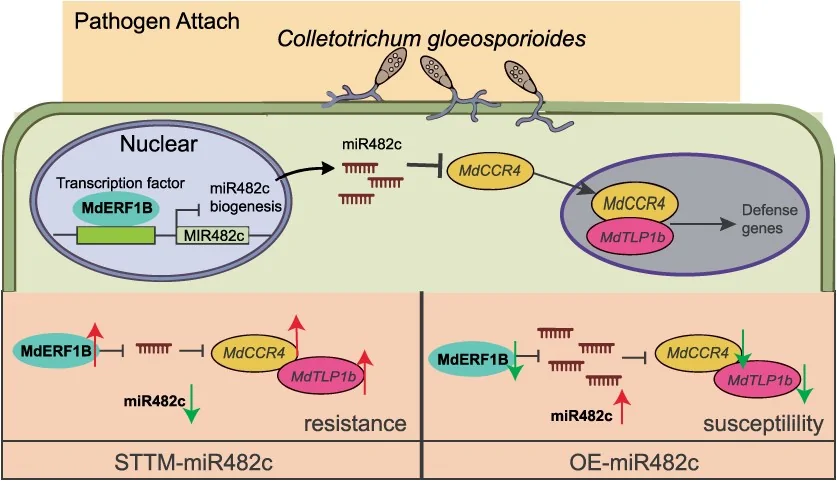
The working model of the MdERF1B-miR482c-*MdCCR4-MdTLP1b* module in apple in response to *C. gloeosporioides* infection.

A growing body of evidence has shown that microRNAs (miRNAs) fine-tune plant immune responses via transcript degradation or translational inhibition [44, 45]. The miR482 family, highly conserved across plant species, is canonically known to target NBS-LRR transcript [8]. For instance, the elevated levels of miR482 in tomato and potato negatively regulate NBS-LRR targets, resulting in impaired immunity against *P. infestans* and *Verticillium dahliae*, respectively [9, 10]. Consistent with these reports, this study reveals that miR482c functions to suppress the apple defense response to *C. gloeosporioides*; OE of miR482c significantly aggravated disease symptoms, while silencing it enhanced resistance (Fig. 2). However, unlike previous studies that predominantly identified NBS-LRRs as targets, we validated a serine/threonine protein kinase, *MdCCR4*, as a direct target of miR482c via 5′ RACE and co-expression assays in *N. benthamiana* (Fig. 1). This finding represents an expansion of the targeting diversity of the miR482 family beyond the canonical NBS-LRR repertoire. In apple, it may also reflect functional diversification adapted to specific pathogen pressures, where miR482c modulates plant immunity not only by regulating R genes but also by dampening kinase-mediated signaling cascades. By cleaving *MdCCR4*, which physically interacts with the thaumatin-like protein MdTLP1b to activate downstream defense responses (Fig. 4), miR482c adds a novel post-transcriptional regulatory layer to the apple-*C. gloeosporioides* pathosystem. The pathogen-induced upregulation of miR482c in the susceptible cultivar ‘GL-3’ likely reflects a virulence strategy employed by *C. gloeosporioides* to subvert host surveillance by targeting *MdCCR4* (Fig. 1). This broadens our understanding of miR482’s versatile roles in plant-pathogen interactions and underscores species-specific adaptations in woody perennials.

Serine–threonine kinases are pivotal in mediating signal transduction and triggering plant immune responses [46]. A classic example is the tomato Pto kinase, which mediates defense signaling not only by phosphorylating the downstream target Pti1 but also by interacting with the ERF transcription factor Pti4 to directly induce defense gene expression [47–49]. Similarly, in tobacco, NrSTK has been characterized as a defense enhancer against black shank, whose biological function requires the presence of a conserved ATP-binding site as well as a motif essential for serine/threonine kinase activity [50]. Similarly, the introduction of the cotton kinase *GbSTK* into Arabidopsis significantly improved plant tolerance to both *V. dahliae* and oxidative stress through the activation of immune signaling and the maintenance of ROS homeostasis [51]. In our study, the result shows that OE of *MdCCR4* outstandingly confer tolerance to *C. gloeosporioides* (Fig. 3). In an effort to elucidate the underlying mechanism, we searched for MdCCR4-binding proteins and identified MdTLP1b, a thaumatin-like protein. We verified that MdCCR4 physically binds to MdTLP1b in dual assays (*in vivo* and *in vitro*) (Fig. 4), and further demonstrated that the abundance of *MdCCR4* positively modulates the transcript levels of *MdTLP1b* (Fig. S19E). These findings align with recent work in walnut (*Juglans regia*), where the JrCCR4–JrTLP1b interaction was shown to promote anthracnose resistance [52]. To explore the potential for a conserved evolutionary strategy, multiple sequence alignments were performed, revealing high amino acid sequence identity and conserved functional domains between the apple and walnut orthologs (Fig. S20). Collectively, our results underscore that the CCR4–TLP1b interaction represents a conserved defense mechanism across diverse woody plant species.

Thaumatin-like proteins (TLPs), intrinsic members of the PR-5 family, serve as cornerstone components of plant innate immunity, typically functioning by disrupting fungal membrane integrity to inhibit spore germination and mycelial expansion [16, 53]. Aligning with extensive documentation of TLP-mediated resistance in diverse crop species—including rice, wheat, and potato [17, 18, 54], our investigation found that *MdTLP1b* acts as a critical positive regulator of defense against *C. gloeosporioides* in apple (Fig. 5). Specifically, *MdTLP1b* transcription is strongly induced upon infection in the resistant cultivar ‘FJ’ (Fig. S9B) and functional validation assays further found that OE of *MdTLP1b* conferred resistance to *C. gloeosporioides*, whereas RNAi-mediated silencing effectively attenuated the native resistance of ‘FJ’ plants (Fig. 5). Furthermore, a protein sequence alignment revealed that MdTLP1b shares high-sequence identity with the walnut ortholog JrTLP1b (Fig. S20B). These findings strikingly recapitulate the functional characteristics of *JrTLP1b* in walnut, highlighting a conserved antifungal mechanism for TLP1b orthologs in woody perennials combating anthracnose pathogens [52].

The miRNA-target modules exert a vital function in orchestrating plant growth and development [45], and their activities are often fine-tuned by upstream transcription factors (TFs). For instance, the formation of vascular tissues in *Populus* is orchestrated by the PtoTCP20-miR396d-*PtoGRF15* signaling module [55], while in apple, modules such as MdMYB16-miR7125-*MdCCR* [56] and MdBBX22-miR858-MdMYBs [57] coordinate the homeostasis of secondary metabolites. The expression of miRNA-target regulatory circuits during infection is fine-tuned by resistance (R) genes in addition to specific TFs. For instance, the over-accumulation of the R protein SNC1 leads to a global repression of miRNA biogenesis to amplify immune responses [58]. Furthermore, in pear, the transcription factor PcMYB44 associated with PcMIR397 promoter to impair its activity, resulting in relieving the post-transcriptional silencing of laccase genes to promote lignification and broad-spectrum fungal resistance [59]. Similarly, in apple roots, MdERF61 was found to bind specific GCC-boxes within the mdm-miR397b promoter to inhibit its transcription, which subsequently enhances *MdLAC7b*-mediated lignin deposition and confers resistance against *Fusarium solani* [60]. Consistent with these findings, we identified a novel regulatory module, miR482c-*MdCCR4-MdTLP1b*, that is regulated by MdERF1B (Fig. 8). We demonstrated that MdERF1B negatively regulates the transcriptional level of miR482c (Fig. 6), while it also positively modulates apple defense against *C. gloeosporioides* (Fig. 7D–G).

Overall, this study substantiates that the MdERF1B-miR482c-*MdCCR4-MdTLP1b* module orchestrates apple resistance to *C. gloeosporioides*. Specifically, miR482c acts as a negative regulator by post-transcriptionally silencing *MdCCR4*, a serine/threonine protein kinase gene. We determined that MdCCR4 physically interacts with the thaumatin-like protein MdTLP1b, and both components are essential for defense against *C. gloeosporioides*. Moreover, the transcription factor MdERF1B represses miR482c transcription via a direct interaction with its promoter, while upregulation of *MdERF1B* improves resistance to *C. gloeosporioides*.

## Materials and methods

### Pathogen cultures and plant material

*C. gloeosporioides* W16 strain was incubated on PDA medium under 28°C. After making scratch lines 3 d later (to promote conidia production), the culture medium was returned to the incubator for continued cultivation. After continued cultivation for 4 d, the red conidia were harvested using ddH_2_O, followed by straining through four layers of filter to yield a spore suspension. Prior to the inoculation assays, the density of conidial suspension was standardized to 1 × 10^6^ conidia·ml^−1^.

In this study, laboratory-preserved *M. domestica* plantlets, specifically the ‘Royal Gala’ (‘GL-3’) and ‘Fuji’ (‘FJ’) cultivars, were utilized as experimental material. They were propagated on MS within a controlled growth chamber maintained at 25°C with a relative humidity of 80%. The plants were subjected to a 16-h photoperiod with a photon flux density of 120 μmol m^−2^ s^−1^.

Seedlings of *N. benthamiana* were grown in a soil mixture consisting of equal parts of loam, vermiculite, and sterile perlite. The plants were kept in a growth chamber at 25°C with 80% relative humidity.

### Bioinformatics analysis

A genomic fragment of 2 kb located 5′ to the pre-miR482c was downloaded from GDR. For the prediction of putative cis-acting regulatory elements, the sequence was processed using PlantCARE. Subsequently, visualization of these identified motifs was performed with TBtools [61]. Additionally, potential transcription factors (TFs) associating with the miR482c promoter were screened via the ‘Binding Site Prediction’ module in PlantRegMap (http://plantregmap.gao-lab.org/).

### GUS assay

GUS expression levels were assessed through both histochemical staining and quantitative enzyme activity assays, in accordance with the methods outlined by Jefferson *et al.* [62] and Anand *et al.* [63] with minor modifications. For histochemical analysis, the infiltrated leaves were immersed in GUS staining buffer and kept at 37°C. Subsequently, the samples were decolorized using 75% ethanol to completely remove chlorophyll and enhance the visualization of the blue staining.

Spectrophotometric analysis was employed to determine the GUS activity with the aid of a specific assay kit provided by Solarbio Life Sciences (Beijing, China). The assay was conducted in full compliance with the manufacturer’s protocols. The amount of enzyme catalyzing the formation of 1 nmol p-nitrophenol per hour per mg protein was considered as one unit of activity. For each construct, nine independent leaves were infiltrated to ensure statistical reliability.

### 5′ RACE assay

To verify the targeting association between miR482c and *MdCCR4*, the RACE assay was conducted by utilizing the HiScript-TS 5′/3′ RACE Kit as per the instructions provided by the manufacturer. In short, 5′-RACE-ready cDNA was carried out using the supplied 5′ TS Oligo and enzyme mix. Subsequent nested PCR amplification was conducted employing gene-specific outer and inner primer pairs to amplify the cleaved *MdCCR4* transcripts (Table. S2). The obtained PCR products were purified and ligated into a pTOPO-Blunt vector. Finally, randomly selected positive clones were analyzed via Sanger sequencing to map the precise cleavage sites.

### Total RNA extraction and qRT-PCR analysis

Total RNA was extracted from approximately 100 mg of apple leaf tissue employing a column-based purification method [29]. Specifically, the leaf samples were homogenized in 1 ml of lysis buffer supplemented with 150 μl of 10% N-lauroylsarcosine. The homogenate was incubated at 70°C for 10 min, cooled on ice for 5 min, and subsequently centrifuged at 13 000 rpm for 15 min to remove cellular debris. We added 300 μl of pure ethanol to the 600 μl clarified supernatant before transferring the mixture to a spin column. Finally, the RNA was collected in 30–50 μl of RNase-free water after washing.

Subsequently, total RNA was converted into first-strand cDNA utilizing the HiScript II 1st Strand cDNA Synthesis Kit (Vazyme) as per the provided instructions. The RT-qPCR experiments were conducted on a CFX Connect™ Real-Time System (Bio-Rad, Hercules, CA, USA). Finally, relative gene expression levels were quantified using the comparative 2^−ΔΔct^ method [64].

To determine the relative fungal biomass, we followed the protocol of Jiang *et al.* [65] (slightly modified). Genomic DNA extraction from the infected leaves (72 HPI) was carried out with a Kit provided by Sangon Biotech. Data normalization was achieved by using *C. gloeosporioides β-tubulin* gene and *M. domestica MdActin* gene as internal standards.

### *Agrobacterium*-mediated transient transformation

Transformation of *Agrobacterium tumefaciens* GV3101 was achieved via the heat-shock method. Confirmed colonies were cultured in liquid LB broth and cultures were shaken at 180 rpm (28°C) until an OD_600_ of 0.6–1.0 was attained. The harvested cells were resuspended in infiltration buffer. Leaves from 5-week-old tissue-cultured apple plantlets were selected as explants for the transient assay. Infiltration of the *Agrobacterium* suspension was performed using a syringe without a needle. For each construct, 20 independent plantlets were utilized. Following infiltration, the plantlets were maintained at 25 ± 1°C with a 16-h photoperiod before subsequent analysis.

For the *N. benthamiana* assay, the bacterial suspension was standardized to an OD_600_ of 0.8–1.0. Infiltration was carried out on the undersides of leaves from 6-week-old *N. benthamiana* utilizing a syringe without a needle. Postinfiltration, the plants were kept in a climate chamber at 25 ± 1°C.

### Y1H assay

The recombinant pAbAi vector harboring the miR482c promoter was transformed into 50 μl of Y1Hgold yeast cells. Post-transformation, the yeast cells were plated on SD/-Ura and cultured for 3–5 d to screen for positive colonies. Meanwhile, the open reading frames (ORFs) of *Rap2.12*, *Rap2.3*, *ERF9*, *ERF4*, *ERF86*, and *ERF1B* were integrated into the pGADT7 vector to yield *Rap2.12*-pGADT7, *Rap2.3*-pGADT7, *ERF9*-pGADT7, *ERF4*-pGADT7, *ERF86*-pGADT7, and *ERF1B*-pGADT7, respectively. Subsequently, these recombinant plasmids were individually mobilized into the miR482c-promoter-containing Y1H yeast strains. The transformed cells were then cultured on SD/-Leu supplemented with AbA to screen for specific protein–DNA interactions. As a negative control, empty pGADT7 was also transformed into the bait Y1H strains.

### Electrophoretic mobility shift assay

*MdERF1B* ORF was ligated into the pGEX-6p-1 to yield recombinant plasmid GST-*MdERF1B*. Verified positive clones were cultured in LB medium at 37°C for 14 h, followed by culture expansion. When the cell density hit 0.4–0.5, we added 1 mM IPTG to induce protein expression and kept the culture at 28°C for 6 h. The harvested cells were resuspended in PBS (15 ml) and sonicated for 20 min. The lysate was centrifuged, and the supernatant was processed with a GST Fusion Protein Purification Kit (Beyotime Biotechnology) to obtain the target protein. Sangon Biotech (Shanghai, China) provided the 5′-biotinylated probes used for EMSA.

The LightShift Chemiluminescent EMSA kit was employed to conduct the EMSA, following procedures outlined by the manufacturer. First, pre-electrophoresis was performed at 100 V to eliminate impurities. For the binding assay, the purified MdERF1B-GST protein was incubated with unlabeled competitor probes in the binding buffer for 10 min at room temperature, followed by the addition of biotin-labeled probes for an additional 20 min. The reaction mixtures (10 μl) were loaded and separated by electrophoresis. Subsequently, the protein–DNA complexes were transferred onto a nylon membrane. The nylon membrane was cross-linked using UV irradiation (120 mJ/cm^2^) for 3 min on both the front and back sides. After blocking for 15 min, the membrane was incubated with a streptavidin-conjugated HRP antibody (1:2000 dilution) for 15 min at room temperature. Following four washing steps (5 min each), the signals were visualized using ECL reagents.

### ChIP-qPCR

We followed the protocol of Li *et al.* [66] to conduct ChIP assays. The ORF of *MdERF1B* was ligated into the pRI101 to yield the 35S:GFP-*MdERF1B*. Apple calli were then transformed with this plasmid, while control experiments utilized transgenic calli carrying the empty pRI101-GFP vector (35S:GFP). For the ChIP analysis, we employed the Biyoung ChIP Assay Kit based on the manufacturer’s guidelines. Briefly, cross-linked chromatin was isolated from the transgenic calli and immunoprecipitated using an anti-GFP antibody to isolate DNA fragments bound to the GFP–ERF1B fusion protein. The recovered DNA was subsequently analyzed by quantitative PCR (qPCR). ChIP-qPCR assays were conducted on a CFX Connect™ Real-Time System using SYBR Premix Ex Taq reagent. The enrichment of DNA fragments was calculated relative to the input DNA, and data were normalized to the control samples.

### Dual LUC

To analysis transcriptional regulation of miR482c by *MdERF1B*, miR482c promoter was ligated into pGreenII 0800-LUC (Pro_miR482c_-LUC) to generate reporter, carrying Renilla luciferase (REN) gene for normalization. Simultaneously, the *MdERF1B* coding sequence was integrated into pGreenII 62-SK (35S:*MdERF1B*) to yield effector, while 35S functioned as the negative control. Subsequently, reporter and effector were transferred into *A. tumefaciens* GV3101 cells carrying pSoup and injected into *N. benthamiana* leaves. At 48 h postinfiltration, luminescence was visualized using a Tanon 5200 Multi CCD system. The enzyme activities of Firefly and RENs were quantified using a Kit provided by Vazyme, and the relative binding activity was determined by normalizing LUC values to REN.

To assess the transcriptional activity of MdERF1B in *N. benthamiana*, the ORF of *MdERF1B* was ligated into the pBD vector (modified from pGreenII 62-SK) to generate the pBD-*MdERF1B* effector. For controls, pBD-VP16 functioned as the positive standard, whereas the empty pBD vector was used as the negative. Following verification, effector and reporter strains were co-infiltrated *N. benthamiana* leaves. At 48 h postinfiltration, luminescence imaging and relative activity assays were conducted as described above.

### Y2H assay

To construct the bait and prey vectors, ORF of *MdCCR4* and *MdTLP1b* were ligated into pGBKT7 and pGADT7, respectively. The resulting plasmids were co-introduced into Y2HGold yeast cells via the standard polyethylene glycol/lithium acetate protocol. For the controls, pGBKT7-53/pGADT7-T was used as the positive standard, while pGBKT7-lam/pGADT7-T functioned as the negative. Initially, transformants were screened on SD/-Leu/-Trp. To verify protein–protein interactions, yeast cells were transferred to SD/-Ade/-His/-Leu/-Trp containing X-α-gal and cultured at 29°C to observe blue colony formation.

### Pull-down assay

To generate *MdTLP1b*-HIS and *MdCCR4*-GST vectors, the *MdTLP1b* CDS (lacking the stop codon) and *MdCCR4* CDS (lacking the signal peptide and stop codon) were ligated into pET-32a-c and pGEX-6p-1, respectively. These plasmids were then transformed into *E. coli* BL21 (DE3) and *Rosetta* (DE3) cells, and protein expression was induced using IPTG. After induction, the recombinant proteins were purified using the corresponding His- and GST-tag purification kits (Beyotime Biotech). For pull-down assay, the purified *MdTLP1b*-HIS and *MdCCR4*-GST proteins were co-cultured at 4°C for about 2 h. Subsequently, the protein mixture was incubated with His-tag purification resin (Beyotime Biotech) for another 2 h at 4°C. After washing five times to remove unbound proteins, the bound proteins were eluted and subjected to Western blotting analysis using anti-HIS and anti-GST antibodies.

### Firefly luciferase complementation imaging assay

We carried out LCI assay following the method established by Zhao *et al.* [67]. The ORF of *MdCCR4* and *MdTLP1b* was ligated into pCAMBIA 1300-nLUC and pCAMBIA 1300-cLUC to generate nLUC-*MdCCR4* and cLUC-*MdTLP1b*. Using *Agrobacterium*-mediated transformation, we delivered these constructs into the leaves of *N. benthamiana*. Prior to infiltration, various combinations of the transformed *Agrobacterium* strains were suspended in MES buffer at an OD_600_ of 0.8–1.0. After being kept in the dark for 24 h postinfiltration, the plants were moved to a growth chamber and cultured under light conditions for a further 48 h. To detect the interaction, samples were treated with a 1 mM solution of D-luciferin (Promega), subsequently luminescence imaging and relative activity assays were conducted as described above.

### Bimolecular fluorescence complementation assay

To construct BiFC vectors, *MdCCR4* and *MdTLP1b* were integrated into pEarleyGate201-Y_N_ and pEarleyGate202-Y_C_, producing Y_N_-*MdCCR4* and Y_C_-*MdTLP1b*, respectively. Following sequence confirmation, these plasmids were transferred into *A. tumefaciens* GV3101 strain. The harvested cells were suspended using infiltration buffer to achieve an optical density of 1.5. Suspensions of the two strains were combined and co-injected into the leaves of *N. benthamiana*. At 48 hours postinfiltration, confocal images of YFP fluorescence were acquired using a Leica TCS SP8 system (Leica, Germany).

### Subcellular localization

To generate the *MdERF1B*-GFP and *MdCCR4*-GFP fusion construct, the *MdERF1B* and *MdCCR4* coding region (with the stop codon removed) were integrated into pRI101-GFP, whereas empty pRI101-GFP was utilized as negative control. Following verification, plasmids were mobilized into *A. tumefaciens* (GV3101) by heat shock. For transient expression, *Agrobacterium* strains harboring MdERF1B-GFP or the EV were individually mixed with a nuclear marker (mCherry) and co-infiltrated into *N. benthamiana* leaves. GFP and mCherry fluorescence signals were subsequently captured using a confocal laser scanning microscope (Leica TCS SP8, Leica, Germany).

### Analysis of transcriptional activation

To analyze the transcriptional activity of MdERF1B, the ORF of *MdERF1B* was ligated into the pGBKT7 to generate the pGBKT7-*MdERF1B*. As negative and positive controls, the empty pGBKT7 vector and pGBKT7-VP16 were selected, respectively. The constructs were transformed into the yeast strain Y2HGold. The transformed yeast cells were initially selected on SD/-Trp medium. Subsequently, the yeast cultures were cultured in SD/-Trp/-Ade/-His and SD/-Trp/-His/-Ade plates supplemented with X-α-Gal (CX11922, Coolaber, Beijing, China). The plates were incubated at 30°C for 3 d, and the growth status and α-galactosidase activity (blue color development) were photographed.

### AI-based virtual screening for MdCCR4-interacting proteins

To identify potential interacting partners of the serine/threonine protein kinase *MdCCR4*, an AI-based virtual screening was performed following the method established by Li *et al.* [68]. Briefly, the 3D structure of the serine/threonine kinase *MdCCR4* was modeled using AlphaFold. Subsequently, AI-driven virtual screening for MdCCR4-interacting proteins was performed via HDOCK. Proteins with a docking score below −300 were further subjected to AlphaFold-based interaction prediction. Finally, proteins with an ipTM + pTM score greater than 0.75 were selected as potential candidate proteins.

### Statistical analysis

Data analysis was carried out using GraphPad Prism version 9 (GraphPad Prism, San Diego, CA, USA). All assays were performed three times independently, and values are shown as mean ± SD. Differences were assessed by one-way ANOVA (Tukey’s test). Asterisks indicate significance: *P* < 0.05 (^*^), *P* < 0.01 (^**^), and *P* < 0.001 (^***^).

## Supplementary Material

Web_Material_uhag199

## Data Availability

All data supporting this study are included in the manuscript and Supplementary Materials.
