# Supplementary material for: The MdERF1B-miR482c-MdCCR4-MdTLP1b module regulates apple resistance to Colletotrichum gloeosporioides
Source: Hortic Res. 2026 May 21;13(10):uhag199. doi: 10.1093/hr/uhag199 (PMC13633095; doi:10.1093/hr/uhag199)
Supplement: Supplementary file 1 [file web_material_uhag199.zip › Supplementary Figure.pdf]

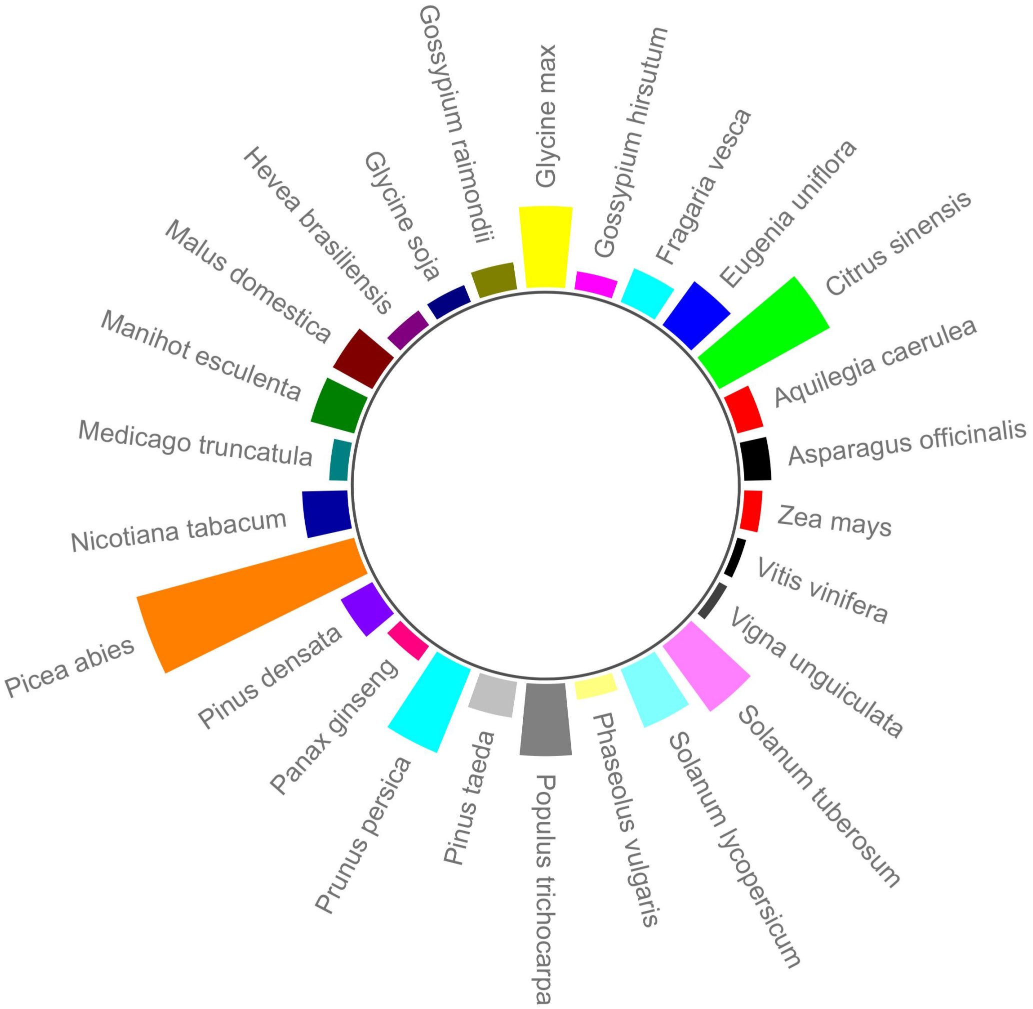

Fig.S1 Statistics of plant miR482 sequences

The miR482 family members were counted in the miRBase database. The column height represents the quantity.

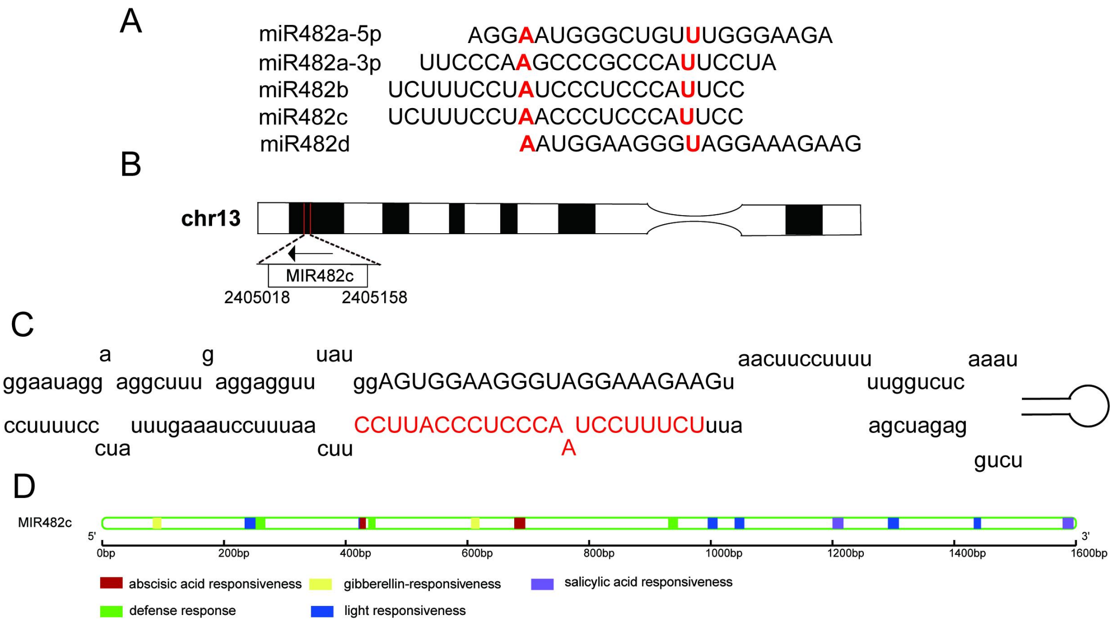

Fig.S2 Analysis of miR482c

A.Sequence alignment of mature miR482 in apple. Red indicates that all bases are identical. B. Genomic localization of the miR482c precursor. C. Predicted secondary structure of the miR482c precursor. The mature miR482c sequence is marked in red. D. Predicted cis-acting elements in the miR482c promoter.

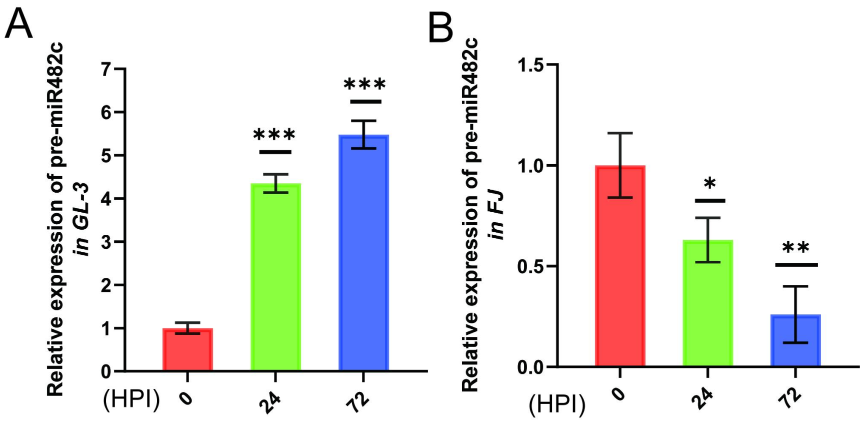

Fig.S3 The expression pattern of pre-miR482c in resistant and susceptible varieties

A.The expression patterns of pre-miR482c in the susceptible varieties 'GL-3'. B.The expression patterns of pre-miR482c in the resistant varieties 'FJ' Asterisks denote statistically significant differences (* $p < 0.05$, ** $p < 0.01$, *** $p < 0.001$). This was determined by one-way ANOVA followed by Tukey's test.

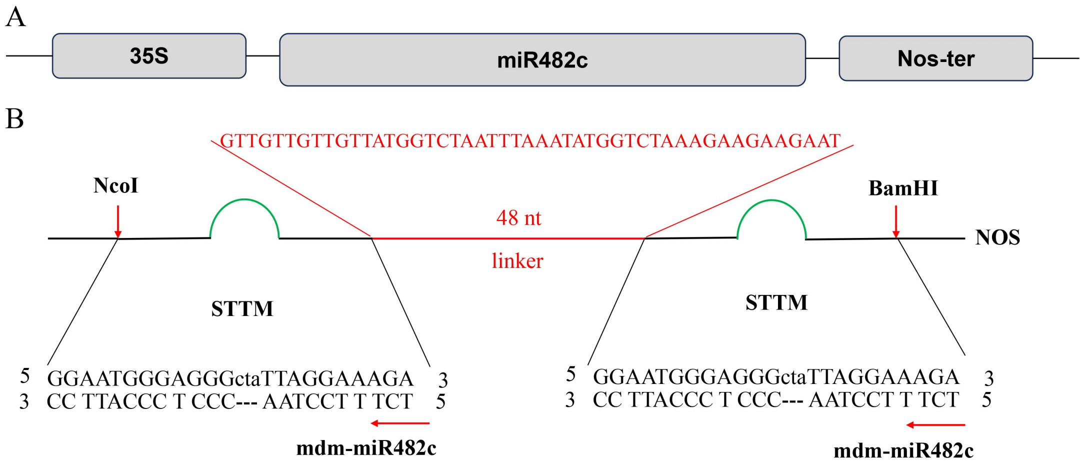

Fig.S4 Schematic diagram of the miR482c vector

A. Overview of the miR482c overexpression construct; B: Schematic illustration of the STTM-miR482c construct.

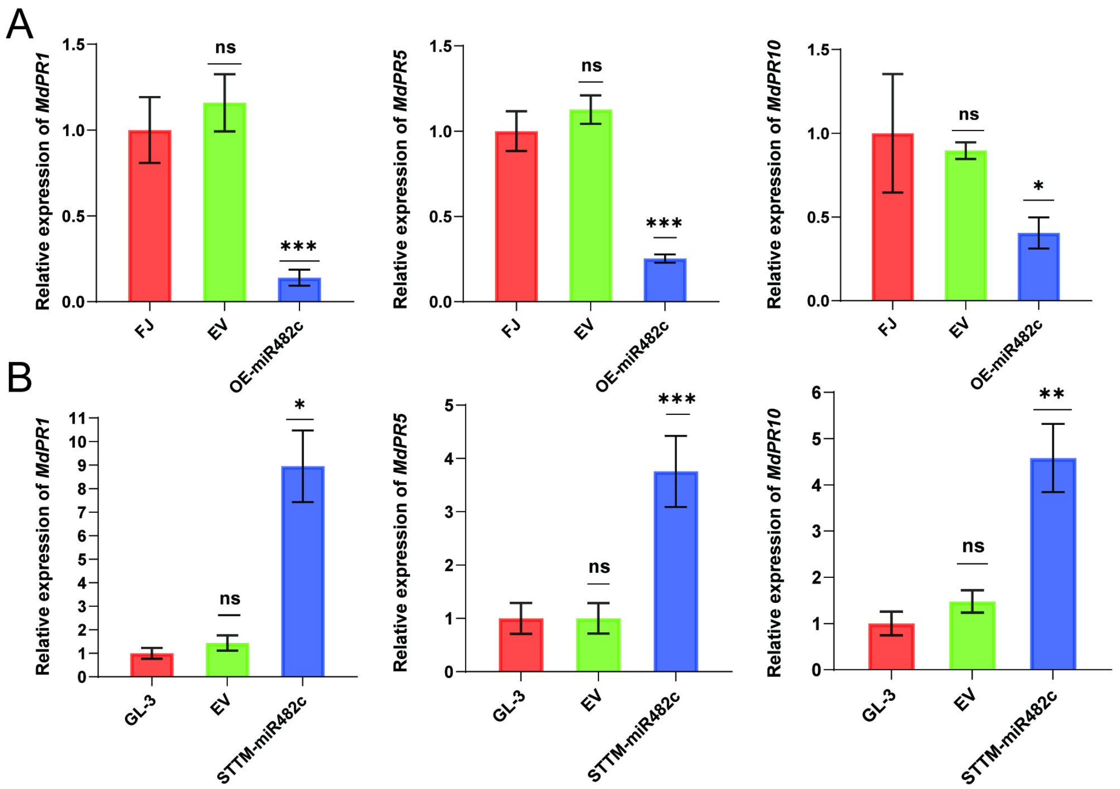

Fig.S5 miR482c affects the expression of pathogen defense-related genes

A. Overexpression of miR482c reduces the expression levels of pathogen defense-related genes *MdPR1*, *MdPR5*, and *MdPR10*. B. Silencing miR482c increased the expression levels of pathogen defense-related genes *MdPR1*, *MdPR5*, and *MdPR10*. Asterisks denote statistically significant differences (* $p < 0.05$, ** $p < 0.01$, *** $p < 0.001$). This was determined by one-way ANOVA followed by Tukey's test.

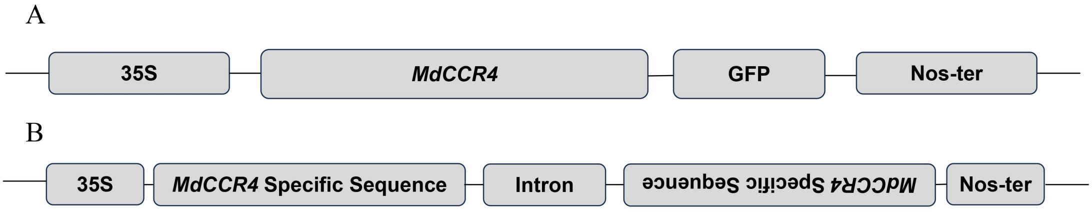

Fig.S6 Schematic representation of the constructs used for *MdCCR4* functional analysis.

A. Schematic structure of the pRI101-GFP vector overexpressing *MdCCR4*. B. The RNAi construct features an inverted repeat of the *MdCCR4* specific sequence separated by an intron spacer.

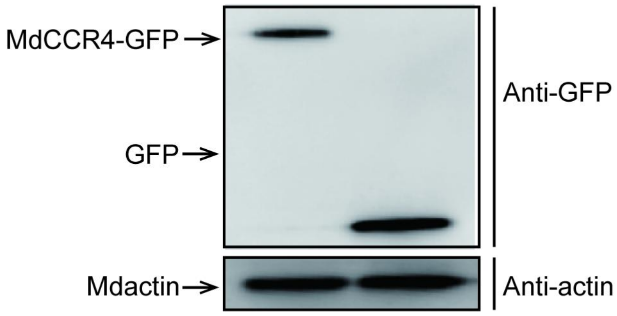

Fig.S7 Immunoblot analysis of the transiently overexpressed MdCCR4 protein in apple leaves.

Total proteins were extracted from 'GL-3' leaves transiently transformed with the empty vector (GFP) or the 35S:MdCCR4-GFP construct at 4 days post-infiltration. The accumulation of the MdCCR4-GFP fusion protein was detected by Western blot using an anti-GFP antibody. The apple MdActin protein was detected with an anti-actin antibody and served as the uniform loading control.

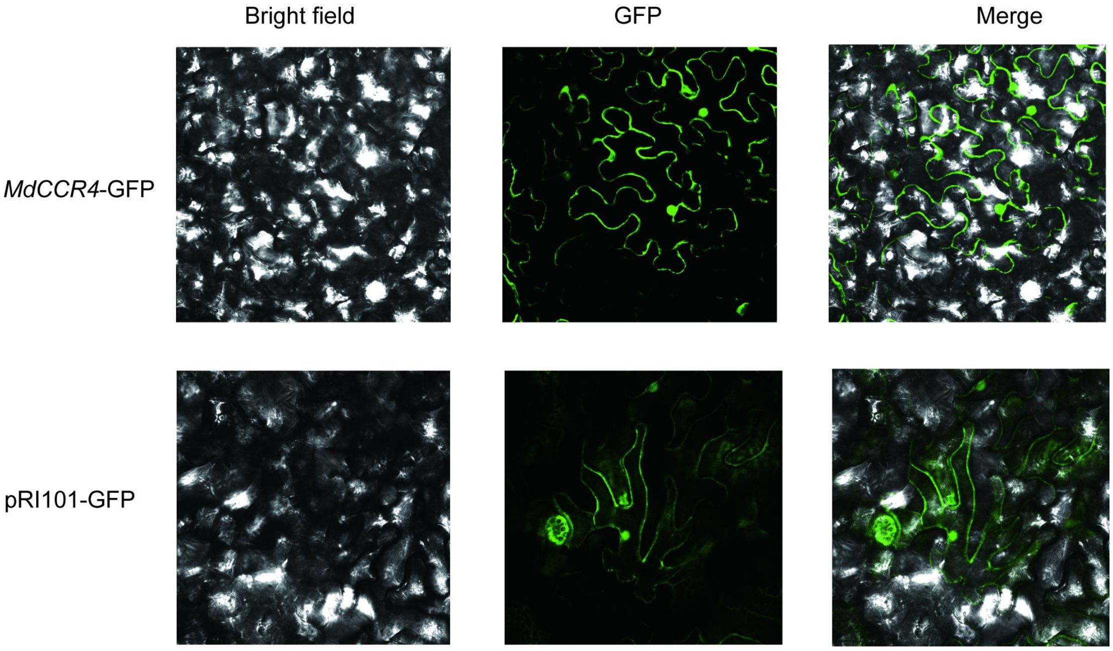

Fig.S8 Subcellular localization of *MdCCR4.*

The *MdCCR4*-GFP fusion protein and pRI101-GFP control were transiently expressed in *Nicotiana benthamiana* leaf cells.

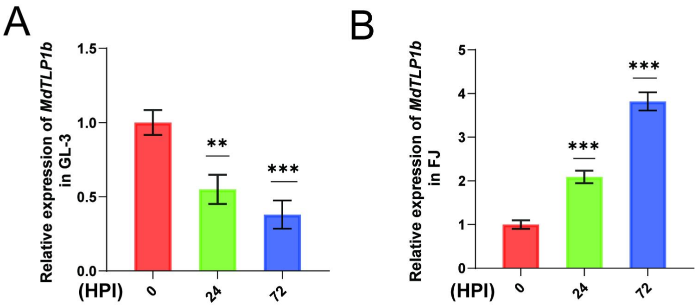

Fig.S9 Expression profiles of *MdTLP1b* in resistant and susceptible cultivars.

Comparative analysis of *MdTLP1b* transcript levels in the susceptible cultivar 'GL-3' (A) and the resistant cultivar 'FJ' (B). Asterisks denote statistically significant differences (* $p < 0.05$, ** $p < 0.01$, *** $p < 0.001$). This was determined by one-way ANOVA followed by Tukey's test.

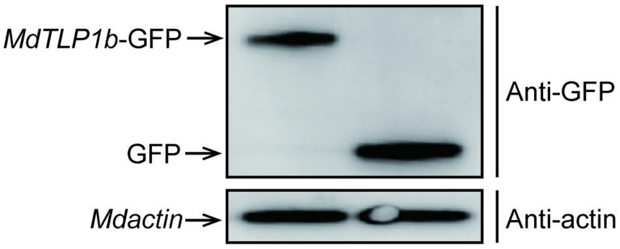

Fig.S10 Immunoblot analysis of the transiently overexpressed MdTLP1b protein in apple leaves.

At 4 days post-infiltration, protein extracts were prepared from 'GL-3' leaves that had been transiently transformed with either the GFP

control vector or the 35S:MdTLP1b-GFP plasmid. The accumulation levels of the recombinant MdTLP1b-GFP protein were evaluated via Western blotting with a GFP-specific antibody. To confirm uniform sample loading, endogenous MdActin was probed using an anti-actin antibody.

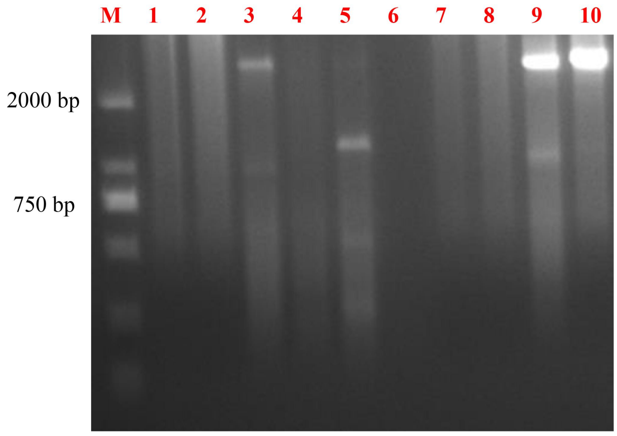

Fig.S11 Cloning of the 2.0-kb genomic fragment upstream of miR482c.

The genomic fragment located upstream of miR482c was amplified by PCR and analyzed via agarose gel electrophoresis. M: DNA molecular weight marker. Lanes 1-10: PCR amplification products.

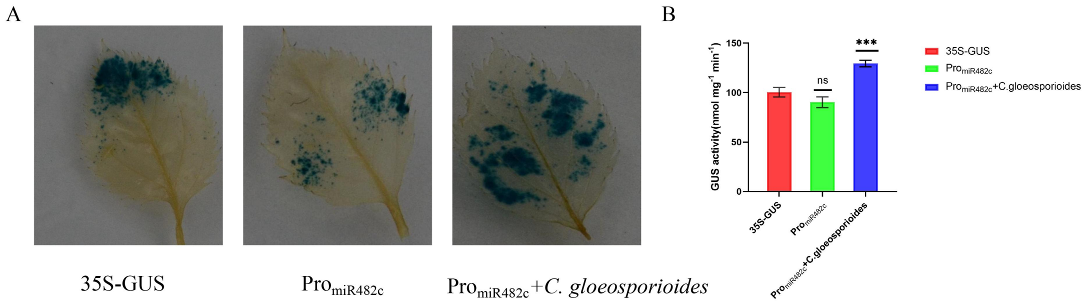

Fig.S12 The activity of the 482c promoter is induced by *C. gloeosporioides* in 'GL-3'

A. Histochemical staining method was used to detect GUS expression in 'GL-3' leaves under different treatments B. UV spectrophotometry was employed to measure GUS enzyme activity. The asterisk indicates a significant difference ($p < 0.001$).

AP2	B3	BBR-BPC	bHLH	C2H2	C3H	CPP	Dof	EIL	ERF	FAR1	GATA	GRAS	HD-ZIP	LBD	LFY	MIKC_MADS	MYB	MYB_related	NAC	Nin-like	SRS	TALE	Trihelix	WRKY
1	5	1	3	3	14	3	59	1	16	1	3	1	2	1	2	22	29	3	3	1	1	2	4	1

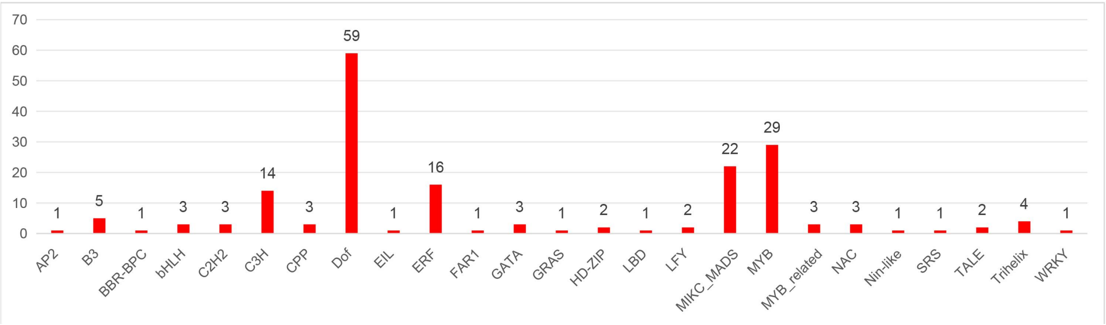

Fig.S13 Prediction of transcription factor binding sites in the 2.0-kb genomic fragment upstream of miR482c.

The 2.0-kb genomic fragment was analyzed to predict potential transcription factors. The table and bar chart display the classification and frequency of transcription factor families predicted to bind to this region.

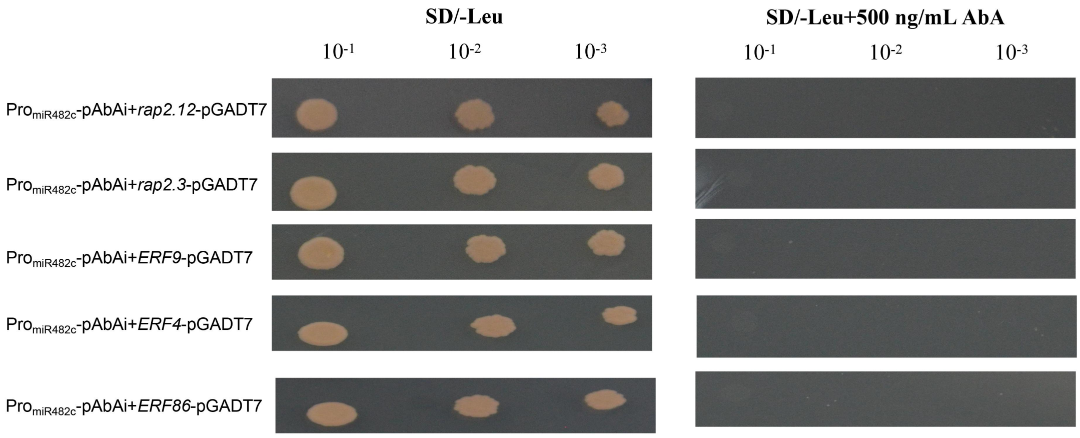

Fig.S14 Y1H assays of other ERF candidates with the miR482c promoter.

Yeast strains carrying the bait vector (PromiR482c-pAbAi) were transformed with prey vectors containing *Rap2.12*, *Rap2.3*, *ERF9*, *ERF4*, or *ERF86*. Transformed cells were spotted on SD/-Leu control medium and SD/-Leu selection medium supplemented with 500 ng/mL AbA at different dilutions (10^{-1} to 10^{-3}).

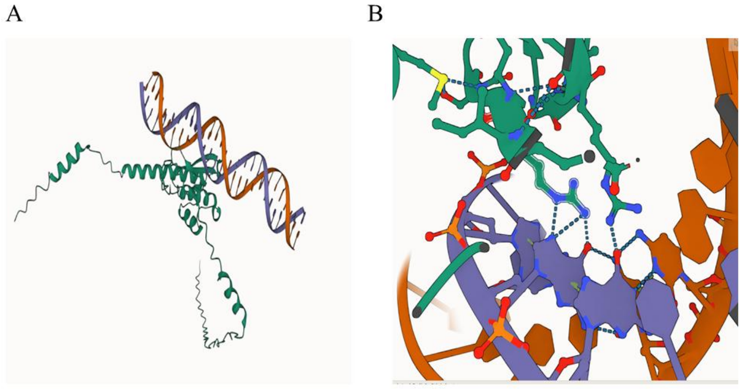

Fig.S15 Molecular docking of MdERF1B with the DNA binding site of miR482c Promoter.

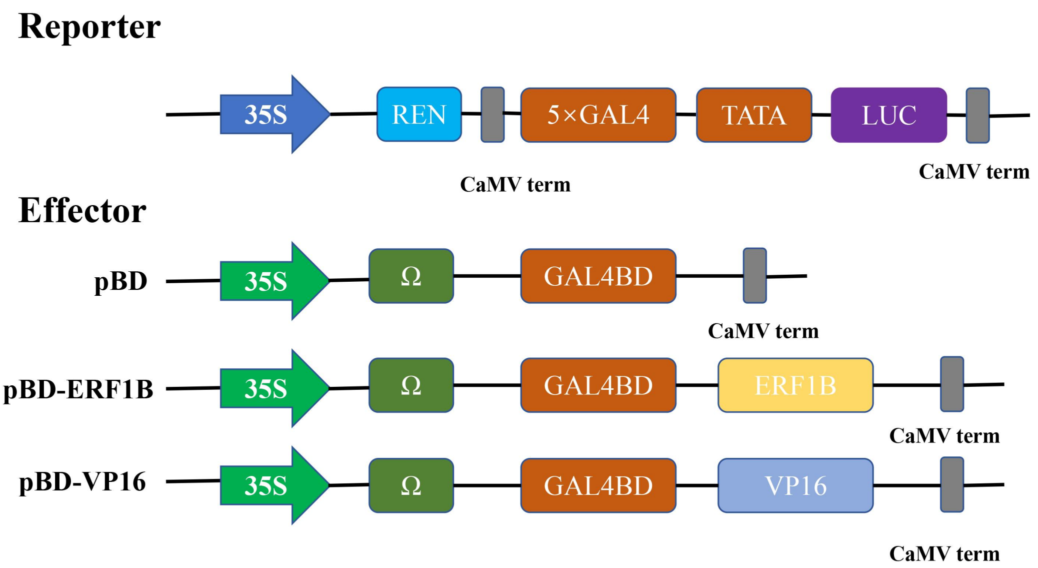

Fig.S16 Schematic representation of the reporter and effector constructs used for the transcriptional activity assay.

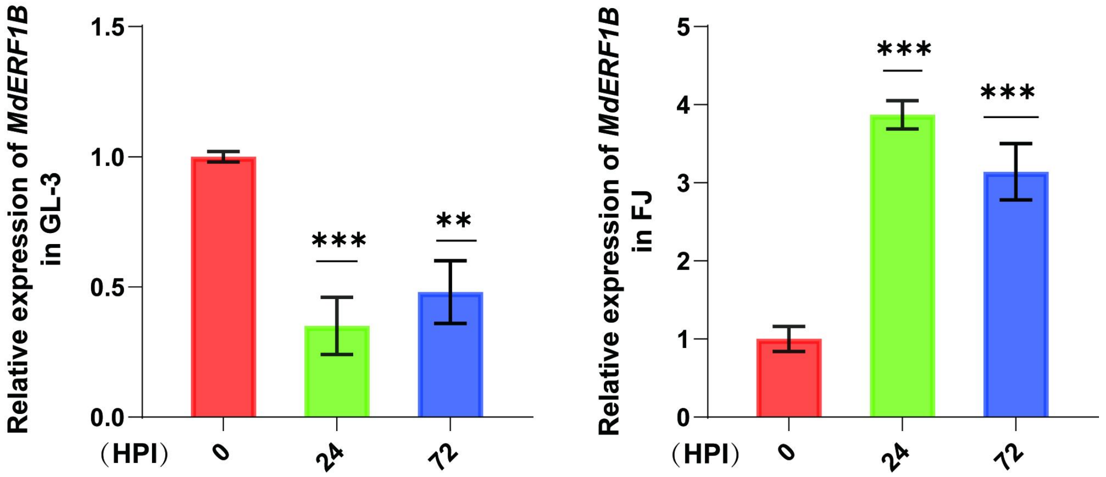

Fig.S17 Expression profiles of *MdERF1B* in resistant and susceptible cultivars.

A. The expression patterns of *MdERF1B* in the susceptible varieties 'GL-3'. B.The expression patterns of *MdERF1B* in the resistant varieties 'FJ' Asterisks denote statistically significant differences (* p < 0.05, ** p < 0.01, *** p < 0.001). This was determined by one-way ANOVA followed by Tukey's test.

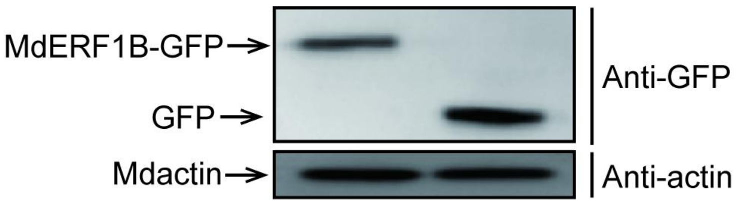

Fig.S18 Immunoblot analysis of the transiently overexpressed MdERF1B protein in apple leaves.

Four days after infiltration, total protein extracts were prepared from 'GL-3' apple leaves transiently expressing either the 35S:MdERF1B-GFP plasmid or the empty GFP vector. Immunoblotting was performed with an anti-GFP antibody to verify the expression of the MdERF1B-GFP fusion protein, while equal protein loading was confirmed using an anti-actin antibody targeting MdActin.

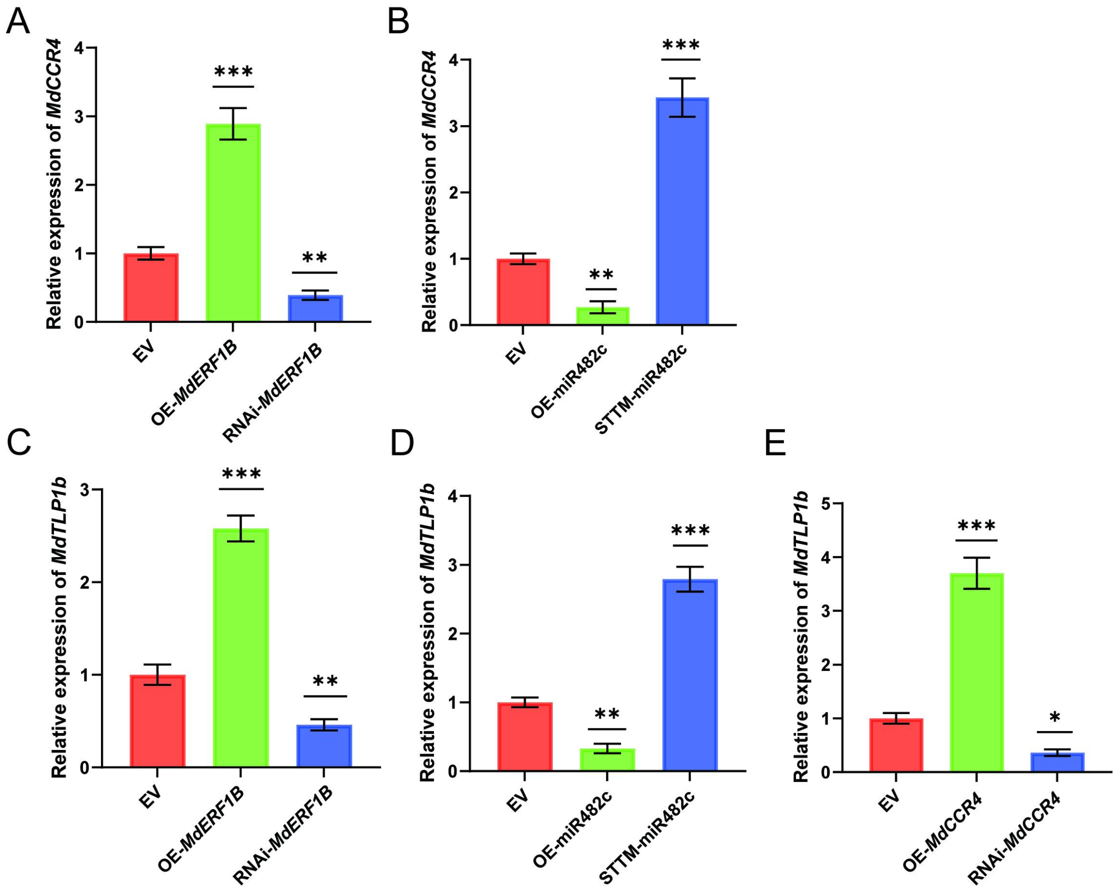

Fig.S19 Expression analysis of downstream genes in the MdERF1B-miR482c-*MdCCR4-MdTLP1b* regulatory module.

(A, B) The relative transcript levels of *MdCCR4* in apple leaves transiently overexpressing or silencing *MdERF1B* (A) and miR482c (B). (C-E) The relative expression levels of the downstream defense gene *MdTLP1b* in apple leaves transiently overexpressing or silencing *MdERF1B* (C), miR482c (D), and *MdCCR4* (E). Empty vector (EV) was used as the control. Total RNA was extracted at 4 days post-infiltration for qRT-PCR analysis. *MdActin* was used as an internal control. Data are presented as means ± SD of three independent biological replicates (n=3). Asterisks indicate significant differences compared to the EV control, determined by one-way ANOVA followed by Tukey's test (* $p < 0.05$, ** $p < 0.01$, *** $p < 0.001$).

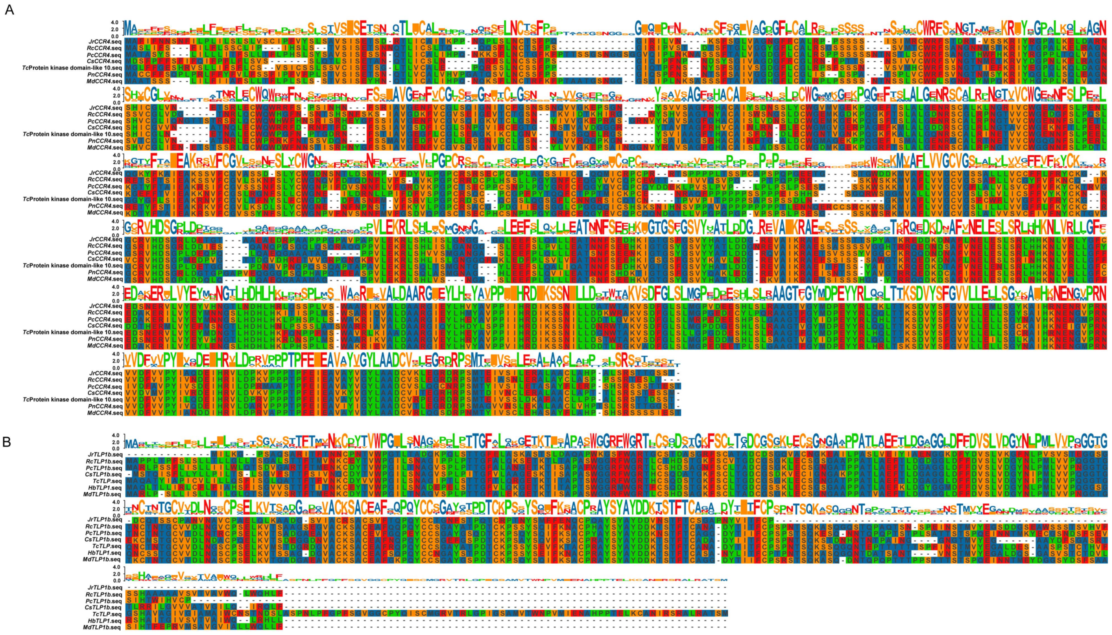

Fig.S20 Multiple sequence alignment and conserved amino acid motifs of MdCCR4 and MdTLP proteins.

The sequence logos above the alignments indicate the conservation level of amino acids at each position, with the height of the letters representing the frequency of the residue. Colored boxes highlight conserved residues across different species. Species abbreviations: Jr, *Juglans regia L.*; Rc, *Rosa chinensis*; Pc, *Pyrus ussuriensis x Pyrus communis*; Cs, *Cucumis sativus*; Pn, *Populus nigra*; Hn, *Hevea brasiliensis*; Tc, *Theobroma cacao*; Md, *Malus domestica*.
